# The increase of long noncoding RNA Fendrr in hepatocytes contributes to liver fibrosis by promoting IL-6 production

**DOI:** 10.1016/j.jbc.2024.107376

**Published:** 2024-05-16

**Authors:** Zhiqian Kang, Chenqi Wang, Fang Shao, Hao Deng, Yanyan Sun, Zhengrong Ren, Wei Zhang, Zhi Ding, Junfeng Zhang, Yuhui Zang

**Affiliations:** 1 State Key Laboratory of Pharmaceutical Biotechnology, School of Life Sciences, Nanjing University, Nanjing, PR China; 2 State Key Laboratory for Organic Electronics and Information Displays (SKLOEID) & Jiangsu Key Laboratory for Biosensors, Institute of Advanced Materials (IAM), Jiangsu National Synergistic Innovation Center for Advanced Materials (SICAM), School of Chemistry and Life Sciences, Nanjing University of Posts and Telecommunications, Nanjing, PR China

**Keywords:** lncRNA, fendrr, hepatocyte, liver fibrosis, STAT2, IL-6

## Abstract

Liver fibrosis/cirrhosis is a pathological state caused by excessive extracellular matrix deposition. Sustained activation of hepatic stellate cells (HSC) is the predominant cause of liver fibrosis, but the detailed mechanism is far from clear. In this study, we found that long noncoding RNA Fendrr is exclusively increased in hepatocytes in the murine model of CCl_4_- and bile duct ligation-induced liver fibrosis, as well as in the biopsies of liver cirrhosis patients. *In vivo*, ectopic expression of Fendrr aggravated the severity of CCl_4_-induced liver fibrosis in mice. In contrast, inhibiting Fendrr blockaded the activation of HSC and ameliorated CCl_4_-induced liver fibrosis. Our mechanistic study showed that Fendrr binds to STAT2 and enhances its enrichment in the nucleus, which then promote the expression of interleukin 6 (IL-6), and, ultimately, activates HSC in a paracrine manner. Accordingly, disrupting the interaction between Fendrr and STAT2 by ectopic expression of a STAT2 mutant attenuated the profibrotic response inspired by Fendrr in the CCl_4_-induced liver fibrosis. Notably, the increase of Fendrr in patient fibrotic liver is positively correlated with the severity of fibrosis and the expression of IL-6. Meanwhile, hepatic IL-6 positively correlates with the extent of liver fibrosis and HSC activation as well, thus suggesting a causative role of Fendrr in HSC activation and liver fibrosis. In conclusion, these observations identify an important regulatory cross talk between hepatocyte Fendrr and HSC activation in the progression of liver fibrosis, which might represent a potential strategy for therapeutic intervention.

Liver fibrosis, characterized by excessive deposition of extracellular matrix (ECM), is a wound-healing response occurring due to chronic liver inflammation or injury (1). The progressive scarring process disrupts the architecture and function of liver, eventually leading to cirrhosis, liver failure, and even hepatocellular carcinoma (2, 3). To date, no antifibrotic therapy has been approved, largely due to the various side effects and limited patient response to the causal treatment, and an incomplete understanding of the pathogenesis (4, 5). Thus, a better understanding of the pathobiology of liver fibrogenic responses is of considerable interest for developing therapeutic strategy against liver fibrosis.

Regardless of etiology, the resident hepatic stellate cells (HSCs) are the principal source of fibrogenic cells and their transdifferentiation into the proliferating, contractile, proinflammatory, and smooth muscle α-actin (α-SMA)-expressing myofibroblasts are responsible for the production of excessive ECM components (6). The activation of quiescent HSC is a result of dynamic interaction between parenchymal and nonparenchymal cells, where the proinflammatory and profibrotic factors are released from various types of resident cells and mediate adverse effects through promoting the activation and proliferation of HSCs. Hepatocytes comprise the bulk of the organ parenchyma and are the most vulnerable to various kinds of insults. In different animal models, as well as various liver diseases, the progression of liver fibrosis is associated with significant hepatocyte injury and the release of various profibrotic factors, including transforming growth factor-beta (TGFβ), connective tissue growth factor, tissue inhibitors of metalloproteinases (TIMPs), and interleukin 6 (IL-6), which are hypothesized to activate the surrounding HSCs in a paracrine manner (7, 8). However, the exact cellular mechanisms involved in the regulation of upstream stimuli from hepatocytes are not well understood.

The long noncoding RNAs (lncRNAs) participate in modulating a wide range of biological processes by various mechanisms related to the cellular location (9). Their involvement in the pathological processes of liver fibrosis have been identified in recent studies. For instance, GAS5 suppresses the activation of HSCs *in vitro* and alleviates the extent of liver fibrosis *in vivo* by acting as a sponge for microRNA-222 (10). The upregulation of H19 is implicated in both bile duct ligation (BDL)- and CCl_4_-induced liver fibrosis through regulating fibrotic gene expression (11, 12). Nevertheless, how dysregulation of the lncRNAs modulates the progression of liver fibrosis is still largely unknown.

The conserved lncRNA Fendrr is transcribed in the opposite direction to the neighboring Foxf1 gene. During embryonic development, Fendrr is specifically detected in the nascent lateral plate mesoderm (13, 14). In the adult, Fendrr is highly expressed in lungs and colon, but almost undetectable in liver (15, 16). Previous studies revealed the dysregulation of Fendrr in adult is implicated in different types of cancer and lung fibrosis as well (17, 18, 19). Mechanistically, Fendrr regulates the target gene expression at transcriptional or posttranscriptional stages. For instance, Fendrr can specifically sequester miRNAs and 3′UTRs of mRNAs as a molecular sponge (20, 21). During the development of lateral plate mesoderm, Fendrr physically binds to PRC2 and TrxG/MLL, the essential chromatin modifying histone methyltransferase complexes, and thus modulates chromatin signatures that define gene activity (14, 22). Nevertheless, whether Fendrr is involved in the pathogenesis of hepatocyte injury and liver fibrosis remains unknown.

In the present study, we observed a significant upregulation of Fendrr in the hepatocytes during liver fibrosis. In animal experiments, knockdown of Fendrr attenuated CCl_4_-induced liver injury and the progression of fibrogenesis, whereas the ectopic expression of Fendrr aggravated the severity of liver fibrosis. Mechanistically, Fendrr enhances the enrichment of STAT2 in the nucleus, and thus promotes the expression and secretion of IL-6 from hepatocytes, which then activated surrounding HSCs in a paracrine manner.

## Results

### Fendrr is increased in hepatocyte in the progression of liver fibrosis

**Figure 1:**
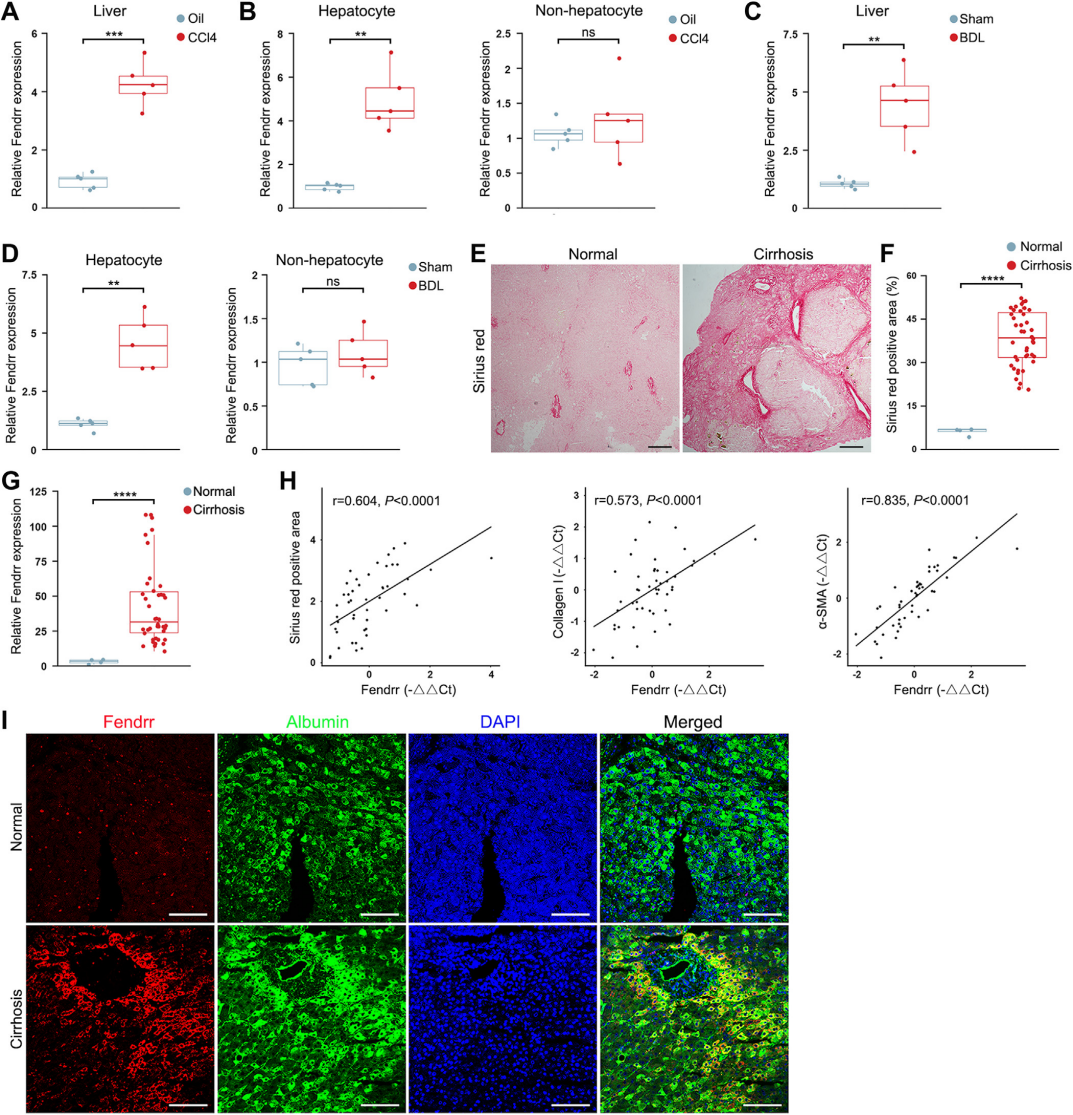
**Fendrr is increased in hepatocyte in the progression of liver fibrosis.***A*, Fendrr expression was determined by qRT-PCR in the CCl_4_-induced murine fibrotic liver (n = 5). The results are shown as fold change compared with oil-treated group (n = 5). *B*, Fendrr is increased in the hepatocytes, but not nonhepatocytes, of the CCl_4_-induced fibrotic liver. *C*, Fendrr expression is increased in the BDL-induced murine fibrotic liver (n = 5). The results are shown as fold change compared with the Sham operation group (n = 5). *D*, Fendrr is increased in the hepatocytes, but not nonhepatocytes, of the BDL-induced fibrotic liver. *E*, representative sirius red staining images of liver cirrhosis patients’ sections. The scale bar represents 100 μm. *F*, quantification of the sirius *red* positive area in the sections from liver cirrhosis patients (n = 41). Nondiseased liver tissue from patients undergoing hepatectomy of hemangiomas was used as normal control (n = 4). Results are expressed as percentage of total area unless noted otherwise in this study. *G*, Fendrr is increased in the specimens from liver cirrhosis patients. The results are shown as fold change compared with the normal control. *H*, scatter plots show the positive correlation of Fendrr with sirius *red*-stained area, and the mRNA expression levels of collagen I and α-SMA in the specimens from liver cirrhosis patients, respectively. The expressions of collagen I and α-SMA mRNA were determined by qRT-PCR. Data points represent measurements of individual patients (n = 41).The Pearson correlation coefficient (r) is shown. *I*, RNA FISH of Fendrr (*red*) and immunofluorescence staining of albumin (*green*) in the specimens from normal control and liver cirrhosis patients. Nuclei were visualized using DAPI staining (*blue*). The scale bar represents 50 μm. For qRT-PCR results, the relative gene expression was normalized against β-actin unless noted otherwise in this study. Data are the mean ± SD of at least three independent experiments. ∗∗*p* < 0.01, ∗∗∗*p* < 0.001, ∗∗∗∗*p* < 0.0001, and ns, no significant change. α-SMA, smooth muscle α-actin; BDL, bile duct ligation; DAPI, 4,6-diamidino-2-phenylindole; qRT-PCR, quantitative real-time PCR.

Given the pivotal role of hepatocytes in liver fibrosis, we isolated the primary hepatocytes from CCl_4_-induced murine liver fibrosis model and performed transcriptome RNA sequencing (RNA-seq) (Fig. S1, *A*–*E*). Analysis of the RNA-seq results showed that 71 lncRNAs were downregulated, and 49 lncRNAs were upregulated in the hepatocytes from fibrotic liver in comparison with the control. Taking the log2 change, Q-value, and fragments per kilobase of transcript per million mapped reads into consideration, Fendrr was one of the most highly upregulated lncRNAs (Fig. S1*F*). To confirm the RNA-seq result, we first examined Fendrr in the CCl_4_-induced fibrotic liver and found that Fendrr is significantly increased after CCl_4_-injection (Fig. 1*A*). We then separated the hepatocytes from other types of cells, showing that Fendrr is significantly increased in the hepatocytes, but not in other cells (nonhepatocytes) of the fibrotic liver (Figs. S1*G* and 1*B*). Similar results were observed in the BDL-induced liver fibrosis model (Figs. S2 and 1, *C* and *D*).

To investigate the clinical relevance, we examined Fendrr expression in the specimens from patients with liver cirrhosis, which were characterized by the increased sirius red staining fibrotic area, higher hepatic hydroxyproline content, and the impairment of liver function (Figs. 1, *E* and *F* and S3, *A* and *B*). By quantitative real-time PCR (qRT-PCR), we found that Fendrr is significantly increased in the individuals with liver cirrhosis in comparison with the normal control (Fig. 1*G*). Of note, there was a positive linear correlation between Fendrr expression and the extent of liver fibrosis, as well as the mRNA expression of α-SMA and collagen I (Col I) in the specimens of liver cirrhosis patients (Figs. 1*H* and S3*C*), suggesting that the increase of Fendrr is clinically involved in the progression of HSC activation and liver fibrosis. Finally, simultaneous RNA FISH to detect Fendrr and immunofluorescence for albumin showed a significant increase of Fendrr in the hepatocytes of liver cirrhosis patients in comparison with the normal control (Fig. 1*I*). Interestingly, Fendrr staining exhibited more enrichment in the necrotic region around central vein area, where the activation of HSCs and the progression of liver fibrosis initiates predominantly, therefore, suggesting a causative role of Fendrr in liver cirrhosis.

### Ectopic expression of Fendrr aggravates CCl_4_-induced liver fibrosis in mice

To investigate the role of Fendrr in liver fibrosis, we generated the lentivirus capable of expressing the full length Fendrr (lv-Fendrr). Injection of the purified lv-Fendrr into mouse *via* tail vein significantly increased Fendrr expression in the liver, but not in the heart, kidney, and lung (Fig. S4*A*). Furthermore, qRT-PCR assay of the isolated hepatocytes revealed that delivery of lv-Fendrr led to the ectopic expression of Fendrr in the hepatocytes, but not in other cells (Fig. S4*B*).

**Figure 2:**
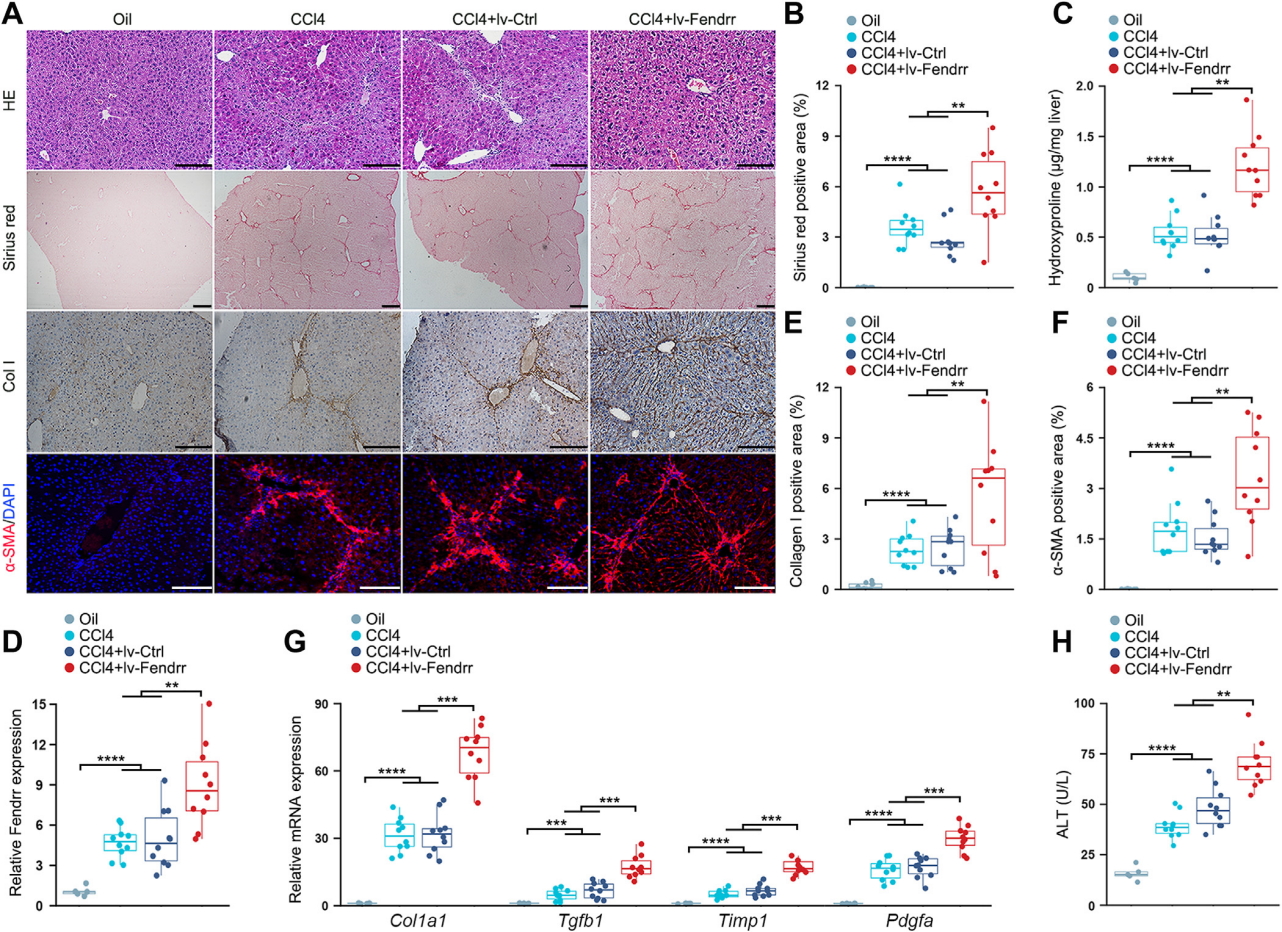
**Ectopic expression of Fendrr aggravates CCl**_**4**_**-induced liver fibrosis in mice.** Mice were injected with oil (oil, n = 5), CCl_4_ (CCl4, n = 10), CCl_4_ in combination with injection of control lentivirus (CCl4+lv-Ctrl, n = 10) and CCl_4_ in combination with injection of lentivirus capable of expressing Fendrr (CCl4+lv-Fendrr, n = 10). The lentivirus was injected once at 2 days before the first CCl_4_ injection. *A*, liver fibrosis was evaluated by H&E staining, sirius red staining as well as collagen I immunohistochemical staining and α-SMA immunofluorescence staining. The scale bar represents 100 μm. *B*, quantification of the sirius red positive area. *C*, quantitative evaluation of hepatic hydroxyproline. The hydroxyproline contents are expressed as μg/mg wet liver weight. *D*, Fendrr expression was examined by qRT-PCR. *E*, quantification of Col I staining area. *F*, quantification of α-SMA staining area. *G*, hepatic *Tgfb1, Pdgfa, Col1a1*, and *Timp1* mRNA expression were examined by qRT-PCR. The results are shown as fold change compared with oil group. *H*, assessment of serum ALT levels. Data are the mean ± SD of at least three independent experiments. ∗∗*p* < 0.01, ∗∗∗*p* < 0.001, ∗∗∗∗*p* < 0.0001. α-SMA, smooth muscle α-actin; ALT, alanine aminotransferase; Col I, collagen I; qRT-PCR, quantitative real-time PCR; TIMP, tissue inhibitors of metalloproteinase.

To address the potential of Fendrr in the CCl_4_-induced liver fibrosis, we intravenously injected lv-Fendrr 2 days before the first injection of CCl_4_ and sacrificed the mice after 4 weeks CCl_4_-treatment. In comparison with the control lentivirus (lv-Ctrl), delivery of lv-Fendrr aggravated the severity of CCl_4_-induced liver fibrosis and augmented the ECM accumulation as demonstrated by H&E and sirius red staining (Fig. 2*A*). Quantitative analysis of the sirius red positive area and hepatic hydroxyproline content confirmed the profibrotic effect of lv-Fendrr *in vivo* (Fig. 2, *B* and *C*). When examining Fendrr, we found that the delivery of lv-Fendrr greatly boosted the increase of hepatic Fendrr in the CCl_4_-treated mouse (Fig. 2*D*). Immunohistochemistry further showed that ectopic expression of Fendrr significantly augmented the accumulation of Col I in the CCl_4_-induced fibrotic liver (Fig. 2, *A* and *E*).

α-SMA^+^ HSCs are the primary producers of ECM during the progression of liver fibrosis. Overexpression of Fendrr greatly increased the number of α-SMA^+^ myofibroblasts in the CCl_4_-induced liver fibrosis model (Fig. 2, *A* and *F*). In addition, the mRNA expressions of profibrotic factors, including TGF-β1, platelet-derived growth factor (PDGF), Col I, and TIMP-1, were markedly upregulated in the fibrotic liver delivered with lv-Fendrr in comparison with the control lentivirus-injected group (Fig. 2*G*). Of note, the increase of serum alanine aminotransferase (ALT) level, a classical marker of liver injury, was also significantly augmented by the ectopic expression of Fendrr in the CCl_4_-injected mouse (Fig. 2*H*).

### Inhibiting Fendrr prevents CCl_4_-induced liver fibrosis in mice

**Figure 3:**
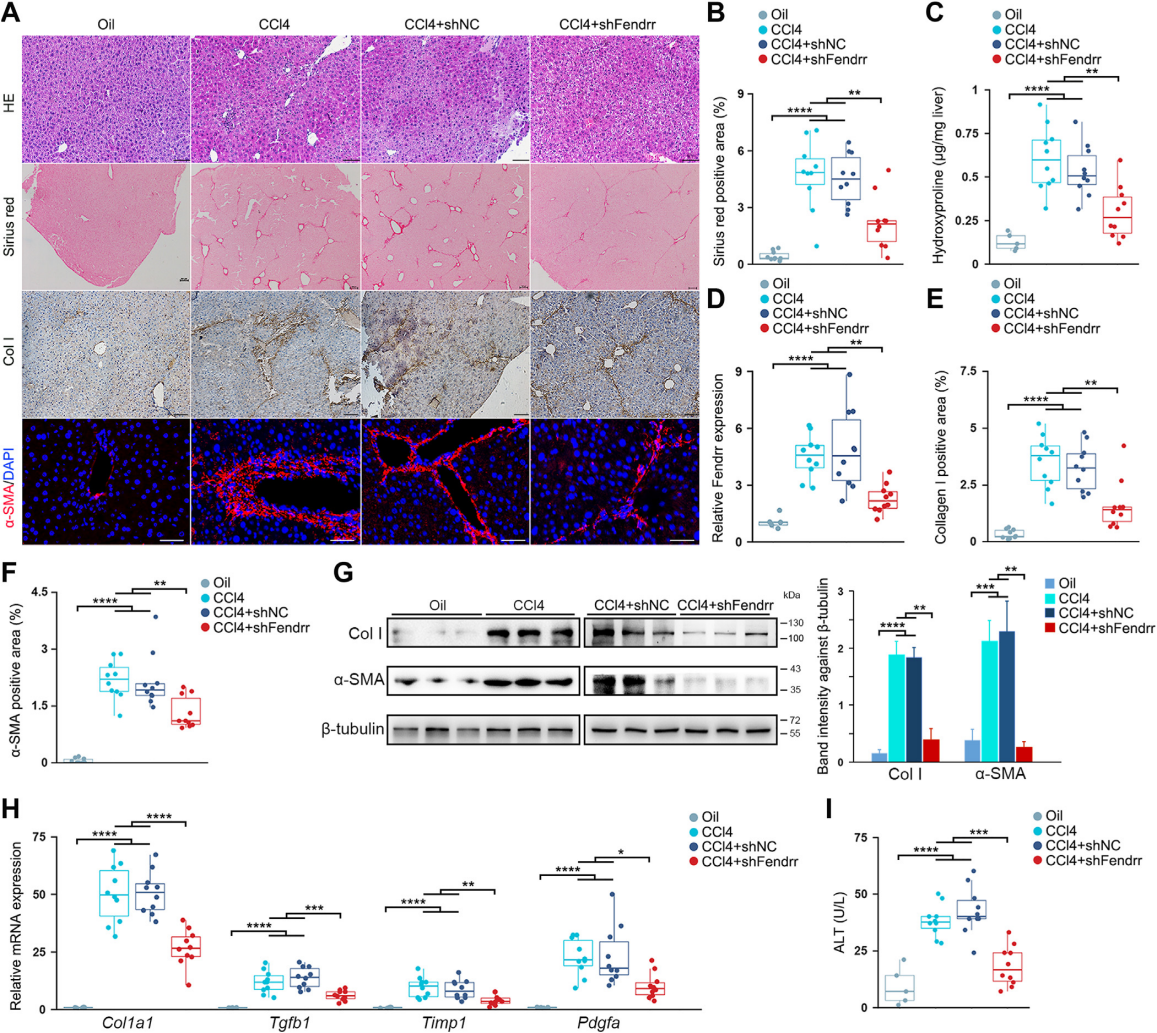
**Knockdown of Fendrr attenuates CCl**_**4**_**-induced liver fibrosis.** Mice were injected with oil (oil, n = 5), CCl_4_ (CCl4, n = 10), CCl_4_ in combination with injection of control lentivirus (CCl4+shNC, n = 10), and CCl_4_ in combination with injection of lentivirus capable of expressing Fendrr shRNA (CCl4+shFendrr, n = 10). The lentivirus was injected once at 2 days before the first CCl_4_ injection. *A*, liver fibrosis was evaluated by H&E staining, sirius red staining as well as collagen I immunohistochemical staining and α-SMA immunofluorescence staining. The scale bar represents 100 μm. *B*, quantification of the sirius red positive area. *C*, quantitative evaluation of hepatic hydroxyproline. The hydroxyproline contents are expressed as μg/mg wet liver weight. *D*, Fendrr expression was examined by qRT-PCR. *E*, quantitative evaluation of Col I staining area. *F*, quantification of α-SMA staining area. *G*, hepatic collagen I and α-SMA were detected by Western blot (*left*). Relative protein level was calculated, respectively, by band intensity against β-tubulin (*right*). *H*, hepatic *Tgfb1, Pdgfa, Col1a1*, and *Timp1* mRNA expressions were examined by qRT-PCR. The results are shown as fold change compared with oil group mice. *I*, knockdown of Fendrr suppresses the increase of serum ALT levels in CCl_4_-treated mice. Data are the mean ± SD of at least three independent experiments. ∗*p* < 0.05, ∗∗*p* < 0.01, ∗∗∗*p* < 0.001, ∗∗∗∗*p* < 0.0001. α-SMA, smooth muscle α-actin; ALT, alanine aminotransferase; Col I, collagen I; qRT-PCR, quantitative real-time PCR; TIMP, tissue inhibitors of metalloproteinase.

To further determine the pathologic contributions of Fendrr to liver fibrosis, we generated the lentivirus capable of ectopically expressing Fendrr specific shRNA (shFendrr). Intravenous injection of the lentivirus into mouse significantly reduced the expression of Fendrr in the liver tissue and the isolated hepatocytes as well (Fig. S5). We then tested whether knockdown of hepatocyte Fendrr would prevent liver fibrosis by intravenously injecting the lentivirus 2 days prior to the first injection of CCl_4._ By 4 weeks of CCl_4_-treatment, the CCl_4_-treated liver displayed a typical feature of fibrosis, whereas delivery of the Fendrr shRNA lentivirus greatly alleviated the severity of fibrosis and reduced the fibrotic area and hepatic hydroxyproline content (Fig. 3, *A*–*C*). Compared to the control virus (shNC), delivery of shFendrr significantly suppressed the increase of Fendrr in the fibrotic liver (Fig. 3*D*). Immunostaining assay and Western blot both demonstrated that inhibition of Fendrr greatly decreased the product of Col I and α-SMA, and reduced the number of α-SMA^+^ myofibroblasts (Fig. 3, *A* and *E*–*G*). Furthermore, knockdown of Fendrr in the CCl_4_-induced fibrotic liver greatly suppressed the upregulation of profibrotic TGF-β1, PDGF, Col I, and TIMP-1 (Fig. 3*H*). Finally, inhibition of Fendrr significantly prevented the increase of serum ALT and improved the liver function (Fig. 3*I*).

### Fendrr interacts with STAT2

**Figure 4:**
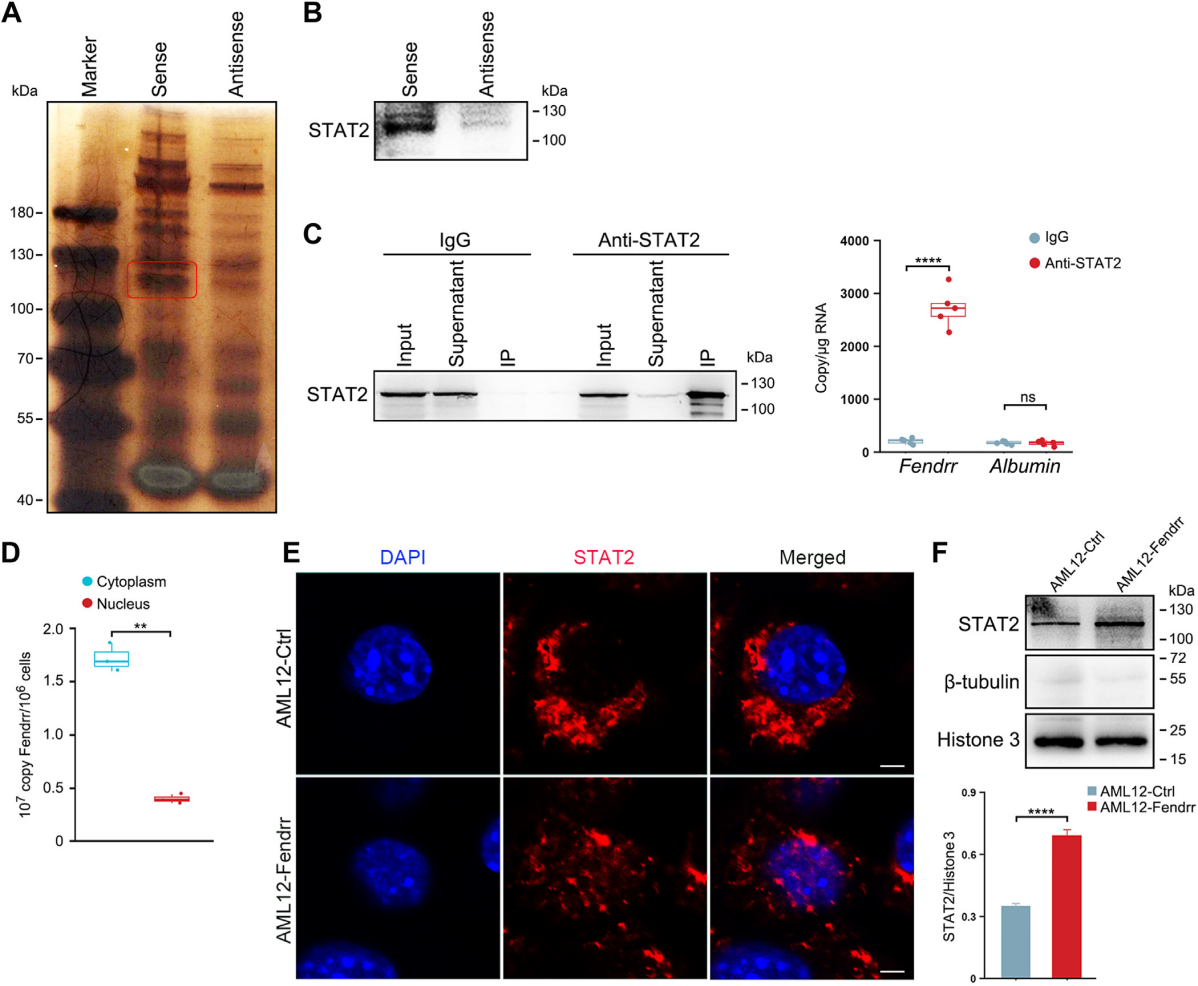
**Fendrr binds to STAT2.***A*, silver staining of proteins bound to Fendrr. The RNA pull-down was performed with AML12 cell lysates. A specific band was identified as STAT2 by mass spectrometry. *B*, the interaction between Fendrr and STAT2 was confirmed by RNA pull-down and Western blot. The biotin-labeled Fendrr and antisense control were mixed with the extracts from AML12 cells, and then incubated with streptavidin agarose beads. The retrieved proteins were assayed by Western blot. *C*, RNA immunoprecipitation (RIP) assay revealed that Fendrr interacts with STAT2. Whole-cell lysates of AML12 cells were immunoprecipitated with STAT2 antibody or IgG. Western blot analysis confirmed IP of endogenous STAT2 from AML12 cells (*left*). qPCR analysis for Fendrr and albumin mRNA in the anti-STAT2 or IgG immunoprecipitates revealed the binding of Fendrr to STAT2 (*right*). The results were shown as the copy number per μg RNA. *D*, subcellular fractionation and quantitative assay of Fendrr copy numbers in the cytoplasm and nucleus of AML12-Fendrr cells. About 1 × 10^6^ AML12-Fendrr cells were collected for subcellular fractionation and RNA purification. The results were shown as the copy number of Fendrr per 10^6^ cells. *E*, Fendrr promotes the enrichment of STAT2 in the nuclei of hepatocytes. AML12-Fendrr cells were stained for STAT2. Nuclei were visualized using DAPI staining (*blue*). The scale bar represents 10 μm. *F*, nuclear fractionation and Western blot assay of STAT2 in the nucleus (*left*). Relative protein level was calculated by band intensity against histone 3 (*right*). Results from at least three independent experiments are shown as mean ± SD. ∗∗∗∗*p* < 0.0001, and ns, no significant change. DAPI, 4,6-diamidino-2-phenylindole; IgG, immunoglobulin G; IP, immunoprecipitation; qPCR, quantitative PCR.

To explore the profibrotic role of Fendrr, we sought to identify the proteins that are associated with Fendrr by an RNA-pull-down experiment, where the *in vitro* transcribed biotinylated Fendrr and an antisense control were mixed with the extracts from the murine hepatocyte cell line AML12, respectively. We resolved the RNA-associated proteins on SDS-PAGE gel and found that an obvious band at approximately 120 kDa was specifically enriched (Fig. 4*A*). Mass spectrometry (MS) analysis of the band identified STAT2 as specifically associated with Fendrr (Table S1). This was then validated by independent Western blot (Fig. 4*B*). Using STAT2 specific antibody, we next performed RNA immunoprecipitation (RIP) and measured the levels of Fendrr in the immunoprecipitates. Compared to immunoglobulin G (IgG) controls, Fendrr, but not albumin mRNA, was significantly enriched in the STAT2-pull-down samples (Fig. 4*C*). Together, these results suggest a specific interaction between Fendrr and STAT2 protein.

To further clarify the mechanism by which Fendrr exerts its effect on liver fibrosis, we generated AML12 subline that stably overexpresses Fendrr (referred to as AML12-Fendrr) using lentiviral vectors (Fig. S6, *A* and *B*). Subcellular fractionation and quantitative assay of the purified cytoplasmic and nuclear RNA from AML12-Fendrr cells showed that Fendrr is predominantly enriched in the cytoplasm (Fig. 4*D*). When examining the effect of Fendrr on STAT2, we found that overexpression of Fendrr did not alter STAT2 content at both mRNA and protein levels in comparison with the control cells (AML12-Ctrl) (Fig. S6, *C* and *D*). Nevertheless, immunostaining assay revealed that Fendrr significantly promoted the enrichment of STAT2 in the nuclei in comparison with the control cells, where the STAT2 protein was predominantly accumulated in the cytoplasm (Fig. 4*E*). To further confirm this observation, we performed subcellular fractionation assays and found that overexpression of Fendrr significantly increased the amount of STAT2 protein in the nuclei of hepatocytes (Fig. 4*F*).

### Hepatocyte Fendrr promotes the activation of HSC through inducing IL-6

**Figure 5:**
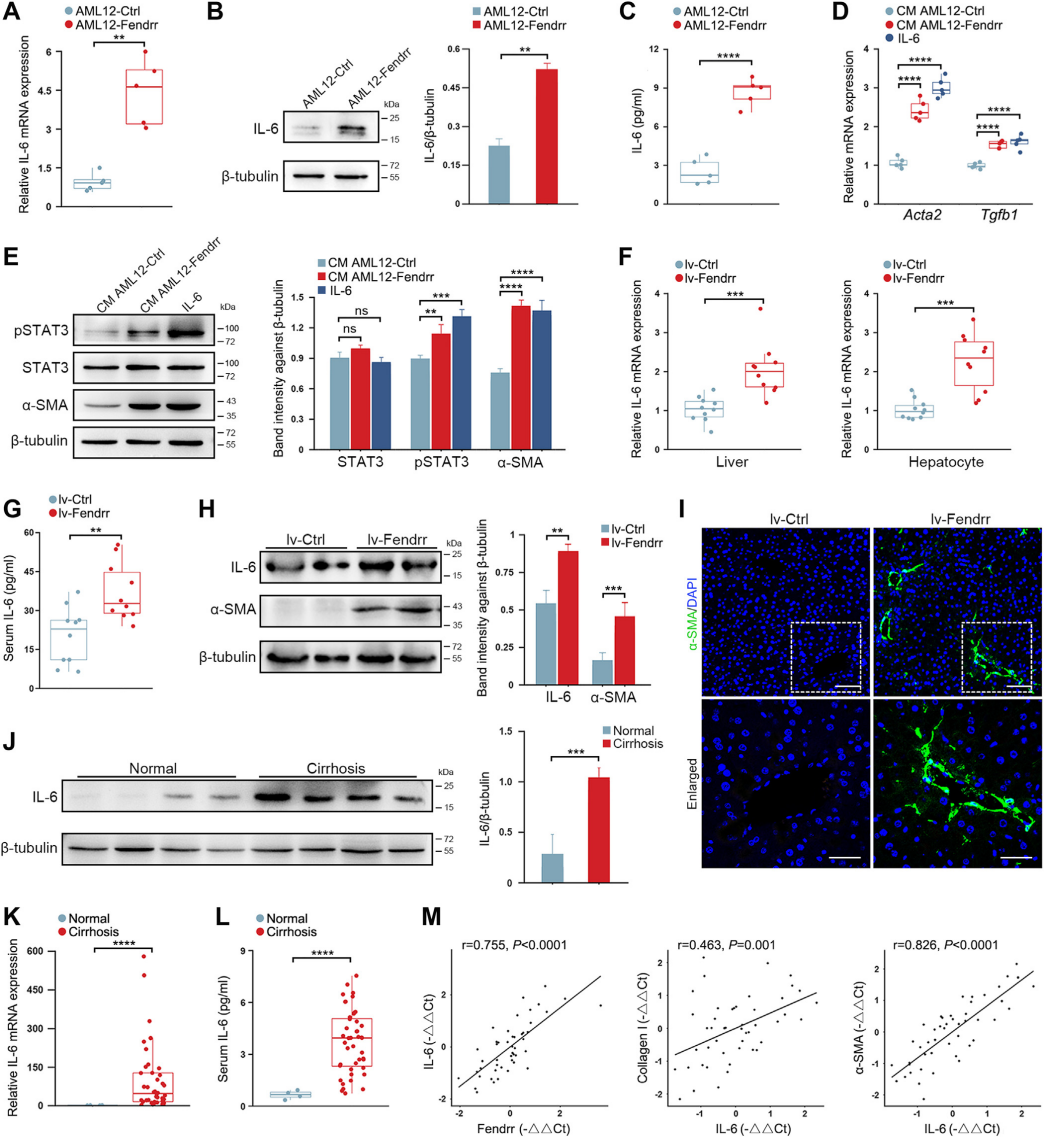
**Hepatocyte Fendrr promotes the activation of HSC in a paracrine manner through enhancing IL-6 expression.***A* and *B*, Fendrr promotes IL-6 expression in AML12 cells. The increase of IL-6 in AML12-Fendrr cells was detected by qRT-PCR (*A*) and Western blot (*B*) respectively. *C*, detection of IL-6 product in the medium of AML12-Fendrr cells by ELISA. *D* and *E*, the CM of AML12-Fendrr cells promotes the activation of HSC. HSC-T6 cells were incubated with IL-6 at a final concentration of 10 ng/ml or the CM from AML12-Fendrr subline for 48 h. TGF-β1 and α-SMA expressions were assayed by qRT-PCR (*D*). STAT3, pSTAT3, and α-SMA were detected by Western blot (*E*). Relative protein level was calculated by band intensity against β-tubulin. *F*, delivery of lv-Fendrr into mouse led to a significant increase of IL-6 mRNA in liver and the isolated hepatocytes. *G*, serum IL-6 is increased in the lv-Fendrr-injected mice. *H*, Western blot assay of IL-6 and α-SMA in the liver of lv-Fendrr-injected mice. *I*, Immunostaining for α-SMA in the liver sections of lv-Fendrr-injected mice. Nuclei were visualized using DAPI staining (*blue*). The scale bar represents *top*, 50 μm; *bottom*, 25 μm. *J*, hepatic IL-6 was detected by Western blot in the specimens from liver cirrhosis patients. *K*, IL-6 mRNA expression was determined by qRT-PCR in the specimens from liver cirrhosis patients (n = 41). Nondiseased liver tissue from the hemangiomas patients was used as normal control (n = 4). *L*, serum IL-6 in liver cirrhosis patients was assayed by ELISA. *M*, the mRNA expression IL-6 is positively correlated with Fendrr, collagen I and α-SMA in the specimens from liver cirrhosis patients (n = 41). The Pearson correlation coefficient (r) is shown. Data are the mean ± SD of at least three independent experiments. ∗∗*p* < 0.01, ∗∗∗*p* < 0.001, ∗∗∗∗*p* < 0.0001, and ns, no significant change. α-SMA, smooth muscle α-actin; CM, conditioned medium; DAPI, 4,6-diamidino-2-phenylindole; HSC, hepatic stellate cell; IL-6, interleukin 6; qRT-PCR, quantitative real-time PCR; TGFβ, transforming growth factor-beta.

Previous study has shown that unphosphorylated STAT2 can constitutively bind to IL-6 promoter and drive the transcription (23). Given the prominent effect of Fendrr on STAT2 enrichment in the nuclei, we hypothesized that the increase of Fendrr might potentially affect IL-6 expression in hepatocytes. As expected, IL-6 was significantly increased in AML12-Fendrr cells at both mRNA and protein levels (Fig. 5, *A* and *B*). ELISA assay also confirmed the increase of IL-6 product in the conditioned medium (CM) (Fig. 5*C*). We then examined IL-6 production by Western blot in the CCl_4_-induced fibrotic livers and found that knockdown of Fendrr substantially suppressed the upregulation of IL-6 (Fig. S7).

IL-6 can robustly promote the activation of HSC by potentiating the phosphorylation of STAT3 (8). Accordingly, exposure of the cultured HSC cell line HSC-T6 to the CM from AML12-Fendrr cells promoted the activation of HSC as indicated by the intensified phosphorylation of STAT3, the upregulation of α-SMA, and TGF-β1, and the increase of migration ability (Figs. 5, *D* and *E*, and S8, *A* and *B*). To substantiate IL-6 as the mediator of fibroblast activation, we added the neutralizing antibody against IL-6 into the CM from AML12-Fendrr cells, and found that administration of the antibody blockaded the profibrotic potentials of the CM (Fig. S8, *C* and *D*). To confirm the potential of Fendrr on HSC activation *in vivo*, we delivered the lentivirus overexpressing Fendrr (lv-Fendrr) into the mice *via* tail vein injection and euthanized the mice 4 weeks later. In line with *in vitro* results, the delivery of lv-Fendrr greatly increased IL-6 expression in liver tissue and the isolated hepatocytes, and thus led to a significant elevation of serum IL-6 concentration (Fig, 5, *F*–*H*). Although one injection of lv-Fendrr did not cause obvious liver fibrosis (data not shown), hepatic α-SMA, the representative marker of HSC activation, was greatly upregulated, suggesting that the increase of Fendrr is implicated with HSC activation, most likely by inducing IL-6 (Fig. 5, *H* and *I*).

Finally, we assayed the hepatic IL-6 expression in human biopsies and found that IL-6 is significantly upregulated in the liver cirrhosis patients as indicated by Western blot and qRT-PCR (Fig. 5, *J* and *K*). Accordingly, serum IL-6 concentration of liver cirrhosis patients is also higher than normal (Fig. 5*L*). Of note, the hepatic Fendrr is positively correlated with IL-6 expression in human biopsies (Fig. 5*M*). Meanwhile, there was a positive linear correlation between IL-6 and the expression of Col I and α-SMA as well (Fig. 5*M*). Similarly, the serum IL-6 concentration positively correlates with the expression of Fendrr, Col I and α-SMA in the specimens of liver cirrhosis patients (Fig. S9). Collectively, these results suggest that the profibrotic role of Fendrr is associated the upregulation of IL-6 and the activation of HSCs.

### The profibrotic role of fendrr depends on its interaction with STAT2

**Figure 6:**
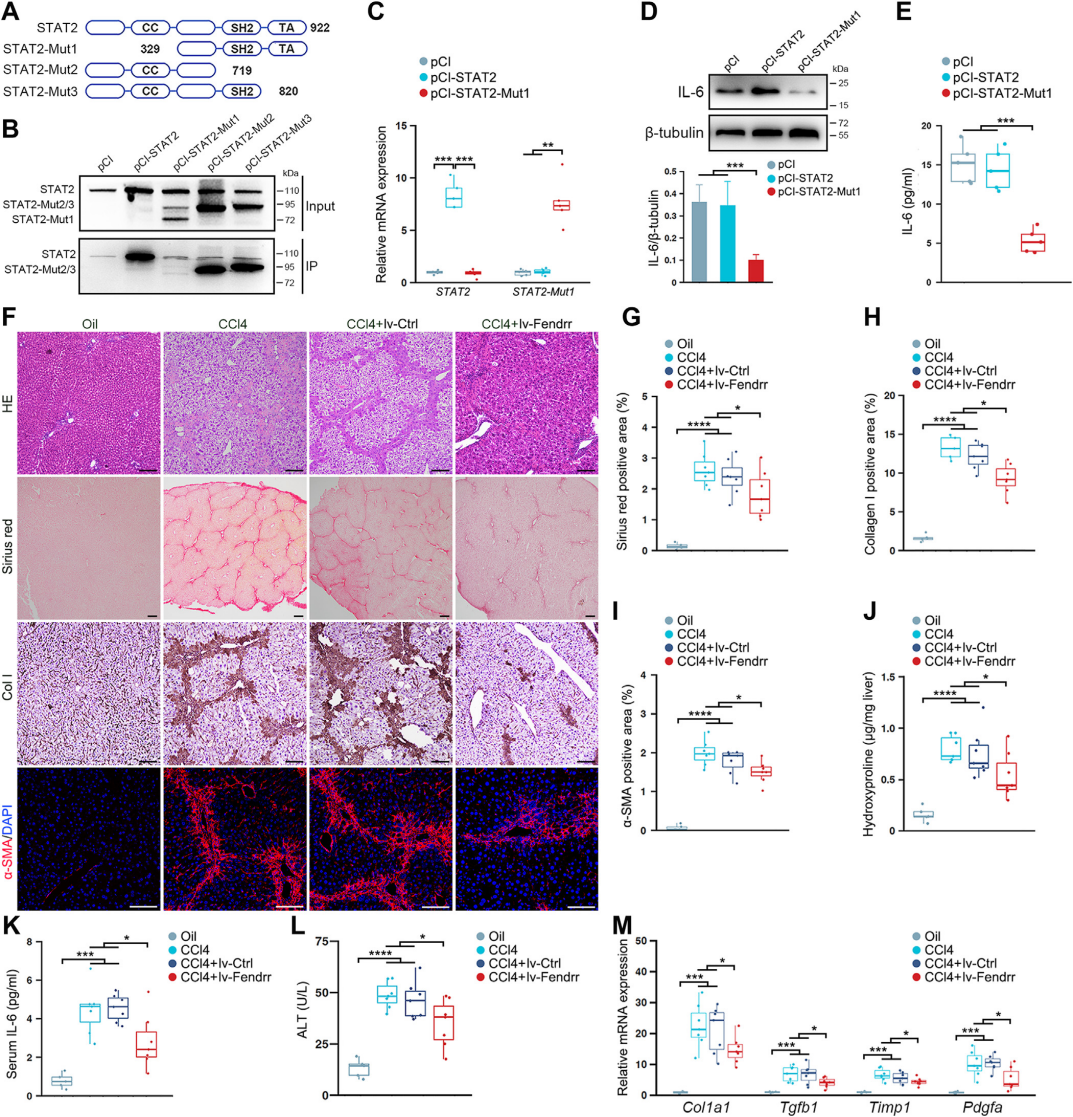
**The profibrotic role of Fendrr in liver fibrosis depends on its interaction with STAT2.***A*, schematic diagram of domain structures of WT STAT2 and STAT2 mutants. *B*, the CC domain of STAT2 is required for its interaction with Fendrr. AML12 cells were transfected with pCI-STAT2, pCI-STAT2-Mut1, pCI-STAT2-Mut2, and pCI-STAT2-Mut3, respectively. The interaction between Fendrr and STAT2 mutant was examined by RNA pull-down using the biotinylated Fendrr and Western blot. *C* and *D*, ectopic expression of STAT2-Mut1 reduced IL-6 expression in hepatocytes. AML12-fendrr cells were transfected with pCI-STAT2 or pCI-STAT2-Mut1 and cultured for 48 h. The expression of STAT2, STAT2-Mut1, and IL-6 were detected by qRT-PCR (*C*) and Western blot (*D*), respectively. *E*, ELISA assay of IL-6 in the medium of AML12-Fendrr cells transfected with pCI-STAT2 or pCI-STAT2-Mut1. *F*, overexpression of STAT2 mutant protect the mice from CCl_4_-induces liver fibrosis. Mice were injected with oil (oil, n = 5), CCl_4_ (CCl_4_, n = 7), CCl_4_ in combination with injection of control lentivirus (CCl4+Ctrl, n = 7), and CCl_4_ in combination with injection of lv-STAT2-Mut1 (CCl4+ STAT2-Mut1, n = 7). The injection of lentivirus was started at 2 days before the first CCl_4_ injection and last for 4 weeks at a frequency of twice per week. The extent of liver fibrosis was evaluated by H&E and sirius red staining, and immunostaining of collagen I and α-SMA. The scale bar represents 100 μm. *G*, quantification of the sirius red positive area. *H*, quantification of Col I staining area. *I*, quantification of α-SMA staining area. *J*, quantitative evaluation of hepatic hydroxyproline. The hydroxyproline contents are expressed as μg/mg wet liver weight. *K*, serum IL-6 was assayed by ELISA. *L*, assessment of serum ALT levels. *M*, hepatic *Tgfb1, Pdgfa, Col1a1*, and *Timp1* mRNA expression were examined by qRT-PCR. The results are shown as fold change compared with oil group mice. *H*, data are the mean ± SD of at least three independent experiments. ∗*p* < 0.05, ∗∗*p* < 0.01, ∗∗∗*p* < 0.001, ∗∗∗∗*p* < 0.0001, and ns, no significant change. α-SMA, smooth muscle α-actin; ALT, alanine aminotransferase; CC, coiled-coil; Col I, collagen I; IL-6, interleukin 6; qRT-PCR, quantitative real-time PCR; TIMP, tissue inhibitors of metalloproteinase.

The full-length murine STAT2 consists of 923 amino acid residues and contains structurally and functionally conserved domains, for instance, the coiled-coil (CC) domain consisting of four α-helices wrapped around each other, the Src-homology 2 (SH2) domain binding to IL-6 promoter element, and the transcriptional activation domain potentiating the transcriptional activity of the target gene (24). To derive a clear picture how Fendrr interact with STAT2, we constructed a series of deletion mutant of STAT2, and overexpressed them in AML12 cells, respectively (Fig. 6*A*). We then mixed the *in vitro* transcribed biotinylated Fendrr with the extracts from AML12 cells overexpressing STAT2 mutant and found that the STAT2 mutant’s lack of N-terminal CC domain failed to interact with Fendrr (Fig. 6*B*). When transferring the plasmid containing STAT2 or the mutant lack of CC domain (referred as to STAT2-Mut1) into AML12-Fendrr cells, we found that the WT STAT2 increased the production of IL-6, whereas ectopic expression of the STAT2-Mut1 significantly suppressed IL-6 expression (Fig. 6, *C* and *D*). ELISA assay further substantiated the inhibitory effect of STAT2-Mut1 on IL-6 production in the medium (Fig. 6*E*). To substantiate the role of STAT2 on IL-6 expression, we transfected AML12-Fendrr cells with STAT2-specific siRNA and demonstrated that knockdown of STAT2 greatly suppressed IL-6 expression and reduced its content in the culture medium (Fig. S10, *A* and *B*). Finally, we performed subcellular fractionation assay, and showed that overexpression of STAT2-Mut1 significantly blockaded the Fendrr-mediated enrichment of STAT2 in the nuclei of hepatocyte (Fig. S10*C*). Collectively, these observations suggest that Fendrr promotes IL-6 expression through its direct interaction with STAT2 and enriching the content of STAT2 in the nuclei.

To test the hypothesis that the profibrotic role of Fendrr depends on its interaction with STAT2 *in vivo*, we generated the lentivirus capable of ectopically expressing the STAT2-Mut1. Injection of the lentivirus into mice *via* tail vein led to a robust expression of STAT2-Mut1 in the liver and hepatocytes as well, whereas the expression of STAT2 was not altered (Fig. S11, *A* and *B*). We then tested whether overexpression of STAT2-Mut1 would prevent CCl_4_-induced liver fibrosis in mice by intravenously injecting the lentivirus 2 days prior to the first injection of CCl_4_. H&E, sirius red staining, and immunostaining of Col I and α-SMA, as well as hepatic hydroxyproline content assay, revealed that ectopic expression of STAT2-Mut1 protected the mice from liver fibrosis (Fig. 6, *F*–*J*). When examining STAT2, we found that the expression of STAT2 did not alter after CCl_4_ treatment and STAT2-Mut1 overexpression (Fig. S11*C*). Of note, injection of the STAT2-Mut1 lentivirus greatly blockaded the increase of serum IL-6 in CCl_4_-treated mice (Fig. 6*K*). These observations were corroborated with the inhibitory effects of STAT2-Mut1 on serum ALT and the expression of profibrotic TGF-β1, PDGF, Col I, and TIMP-1 in the CCl_4_-induced fibrotic liver (Fig. 6, *L* and *M*).

**Figure 7:**
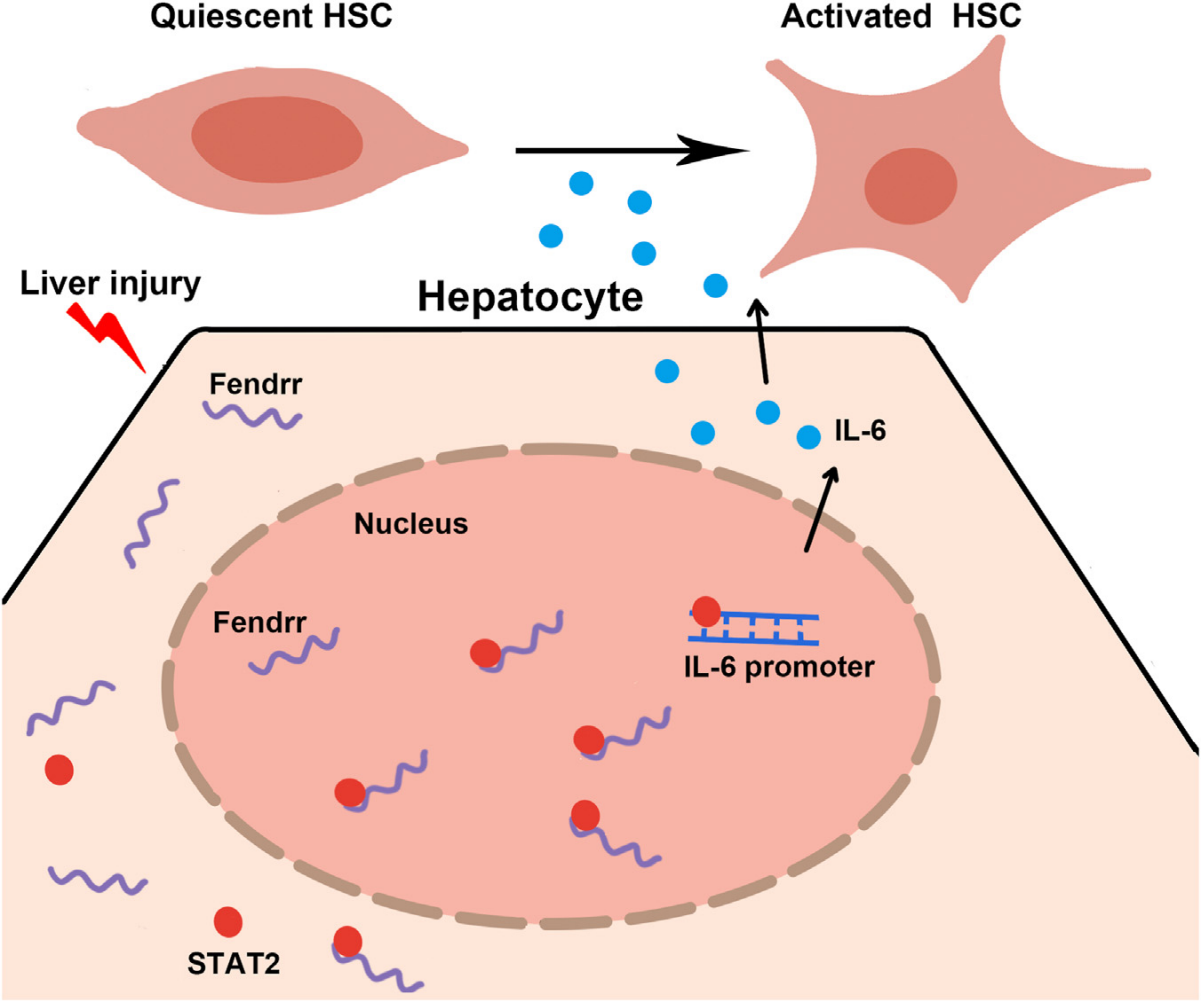
**Schematic diagram showing how increase of Fendrr in hepatocytes promoting the activation of HSC by inducing IL-6.** HSC, hepatic stellate cell.

Finally, we injected the lentivirus simultaneous with lv-Fendrr *via* tail vein at 2 days before the first injection of CCl_4_. As expected, the ectopic expression of STAT2-Mut1 abolished the Fendrr-mediated increase of serum IL-6 in CCl_4_-treated mice and greatly suppressed the profibrotic effect of Fendrr on liver fibrosis (Fig. S12). Collectively, these findings suggested that the causal role of Fendrr in liver fibrosis is associated with its interaction with STAT2, which promotes the induction of IL-6 and activation of HSC in a paracrine manner (Fig. 7).

## Discussion

Liver fibrosis is the hallmark of chronic liver diseases. While transcriptomic profiling and bioinformatics analysis have identified a growing body of lncRNAs associated with the progression of liver fibrosis, experimental validation and characterization of the underlying mechanism are equally important. In the present study, we found that Fendrr is barely detectable in the adult liver, but is exclusively upregulated in the hepatocytes during the progression of liver fibrosis in different animal models and the biopsies of liver cirrhosis patients as well. The progression of liver fibrogenesis is highly dependent on the activation of HSCs, which is predominantly mediated by the profibrotic mediators released from different types of cell, especially the injured hepatocytes. When examining the role of Fendrr in liver fibrosis, we found that the upregulation of Fendrr promoted IL-6 production and secretion from hepatocytes. Previous reports have demonstrated that IL-6 plays a pivotal role in HSC activation (8, 25, 26). Herein, our results indicated that the increase of Fendrr in liver cirrhosis patients is positively correlated with IL-6 production. Meanwhile, the expression of IL-6 positively correlates with α-SMA, the representative marker of HSC activation, in the biopsies of liver cirrhosis patients as well. Considering the important potential of IL-6 in HSC activation, our findings provide mechanistic and translational insights into the causative role of Fendrr in promoting HSC activation and liver fibrosis.

STAT2 is a transcription factor essential for the signal transduction pathway of type I interferons (IFNs), which are known as the classic host immune defense system against viral infections (27). However, the phenotypes of human and murine that are STAT2-deficient have suggested important and currently unidentified type I IFN-independent activities (28, 29). Different from the near-pancellular distribution of the other STAT proteins, STAT2 resides predominantly in the cytoplasm. Upon tyrosine phosphorylation, STAT2 rapidly translocates into the nucleus and acquires the ability to bind specific DNA targets in association with STAT1 and IFN regulatory factor-9 (IRF9) (30). Although the formation of the heterotrimer complex, comprising the phosphorylated STAT1/2 heterodimers and IRF9, is essential to initiate the transcription of STAT2-regulated genes, the unphosphorylated STAT2 can constitutively associate with IFN-stimulated response element at IL-6 promoter and drive the transcription (23). The nuclear translocation of STAT2 depends on its constitutive association with IRF9, which contains a bipartite basic nuclear localization signal, but the presence of STAT2 in the nucleus is transitory (31, 32). STAT2 is effectively exported from the nucleus to the cytoplasm due to an intrinsic nuclear export signal at carboxyl terminus, which serves to shuttle the transcription factor from the nucleus and decrease the effects on target gene expression (33, 34). Our results suggested that binding to Fendrr greatly improved the enrichment of STAT2 in the nucleus, therefore, promoting the expression of IL-6. Nevertheless, the detailed mechanism by which Fendrr helps the nuclear trafficking of STAT2 or prevents the shuttling of STAT2 from the nucleus needs to be further investigated.

Accumulating structural and functional evidence indicate that STAT2 is unique among the STAT proteins because of its ability to associate with the non-STAT protein IRF9, which acts as the first step in the cascade leading to nuclear import (31, 35). The CC domain of STAT2 mediates the binding to IRF9 protein (32, 36). Thus, STAT2-Mut1 is supposed to lose the ability of nuclear translocation due to the lack of CC domain. On the other hand, the dimerization of STAT2 is required for binding to IRF9 (37, 38). Actually, unphosphorylated STATs, including STAT2, were generally present as stable heterodimers or homodimers in the cytoplasm (24, 38). The amount of free STAT monomers is very small in living cells regardless of cytokine treatment or not (39, 40). Taking these into account, we hypothesize that overexpression of the CC domain deficient STAT2-Mut1 will interfere with the formation of STAT2-IRF9 complex in a dominant negative manner, and, therefore, abolished the potential of Fendrr in promoting STAT2 nuclear distribution. Our results showed that overexpressing STAT2-Mut1 in AML12-Fendrr cells reduced the Fendrr-mediated enrichment of STAT2 in the nuclei, and, as a consequence, inhibited the upregulation of IL-6. Based on these observations, we further overexpressed STAT2-Mut1 *in vivo* and showed the inhibitory effects on Fendrr-mediated liver fibrosis.

Fendrr is one of the first lncRNAs that has been identified to be essential for the embryonic development of mammalian the heart, lungs, and gastrointestinal system, and its homozygous loss causes embryonic or perinatal lethality (14). In addition to embryogenesis, the dysregulation of Fendrr has been implicated in the pathogenesis of various malignant cancers, most likely, as a tumor suppressor (17, 41). In terms of organ fibrosis, Fendrr is decreased in the fibrotic human lungs, likely due to the increased TGFβ/Smad3 signaling (19, 42). Mechanistically, Fendrr exerts its antifibrotic effect in lung fibrosis by sequestering miR-214, which is critical for the activation of lung fibroblast (19). However, in a mouse fibrotic heart model, Fendrr is increased and plays a profibrotic role by sponging miR-106b, which targets profibrotic Smad3 mRNA for degradation (20). In the present study, we found that the upregulation of Fendrr occurs mainly in hepatocytes during the progression of liver fibrosis. Thus, the functional role of Fendrr in fibrogenesis might be tissue specific and therapeutic manipulation of Fendrr expression would have to be organ or tissue specific.

Hepatocyte injury is the key trigger of almost all types of liver diseases, and initiates a series of events including the recruitment of inflammatory cells, activation of HSCs, and excessive ECM synthesis and deposition (7). In response to injury, hepatocytes alter their gene expression and secretion profile. To date, the mechanisms linking hepatocyte injury and the release of profibrotic mediators remain poorly understood, but appear to be linked to the sustained regeneration of hepatocyte. It is well-known that hepatocyte has a tremendous proliferative capability to repopulate themselves in mouse models of chronic liver disease (43, 44). In adult liver, developmental signaling pathways are tightly restricted, but these pathways are reawakened upon liver injury and assemble a coordinated wound-healing response. Fendrr is a regulatory RNA essential for proper development of tissues derived from lateral mesoderm. Given its pivotal role in embryonic development and rapid increasing in hepatocyte upon liver injury, we reasoned that upregulation of Fendrr might be a transcriptional response to hepatocyte injury and is required for the following process of replenishment. Nevertheless, further studies are required to reveal whether the sustained increase of Fendrr contributes to the pathogenesis of liver fibrosis by inducing proliferation of hepatocyte and creating a regenerative niche, other than promoting the secretion of IL-6.

The genomic region coding for Fendrr transcript is adjacent, approximately 1.7 kb upstream, to the protein-coding Foxf1 gene in the opposite direction. The gene pairing of Fendrr and Foxf1 indicates that their interval region may harbor the putative promoters of Fendrr and Foxf1, and suggests their coregulation and the potential involvement of Fendrr in etiology of some Foxf1-associated disorders. Actually, a previous report have shown that depletion of Foxf1 in lung fibroblasts by siRNA led to a substantial (∼50%) decrease of Fendrr levels (45). Similarly, deletion of Foxf1 from endothelial cells reduced endothelial proliferation and inhibited vascular endothelial growth factor signaling, partly due to suppressing the expression of Fendrr, which is critical for vascular development (46). However, the binding of Foxf1 to the promoters or the enhancer region of Fendrr has yet to be characterized. To date, there have been contradictory reports on the role of Foxf1 in HSC activation and liver fibrosis, probably due to the utilization of different animal model of liver fibrosis or the methods of manipulating Foxf1 expression *in vivo* (47, 48, 49). Thus, the possible cross talk and mutual regulations between Foxf1 and Fendrr in both the physiological state and pathological processes of liver fibrosis remain to be investigated in future.

In summary, we provide compelling evidences that the increase of Fendrr in hepatocytes promotes the activation of HSC in a paracrine manner by inducing IL-6. Mechanistically, Fendrr binds to STAT2 and improve its enrichment in the nucleus, therefore, promoting the expression of IL-6 in hepatocytes. These observations suggest an important regulatory cross talk between hepatocyte lncRNA and HSC activation, which might represent a potential strategy for therapeutic intervention in liver fibrosis.

## Experimental procedures

### Reagents and antibodies

Dulbecco’s modified Eagle’s medium (DMEM), fetal bovine serum and Lipofectamine 2000 were from Invitrogen. Anti-STAT2 (D9J7L, #72604), anti-pSTAT3 (D3A7, #9145), and anti-STAT3 (D1B2J, #30835) antibodies were purchased from Cell Signaling Technology; anti-IL-6 antibody (#DF6087) was from Affinity Biosciences; anti-α-SMA (BM0002) and anti-Collagen I (BA0325) antibodies were from Boster. The neutralizing antibody against IL-6 (MP5-20F3, #A2118) was from Selleck Chemicals. Other reagents were commercially available.

### Cell culture

The nontumorigenic mouse hepatocyte cell line, AML12 was purchased from the Shanghai Cell Bank, Chinese Academy of Sciences (Shanghai, China), and maintained in DMEM/F12 medium (Invitrogen) containing 10% fetal calf serum, 1 × ITS (insulin-transferrin-sodium selenite media supplement, Sigma-Aldrich), dexamethasone (40 ng/ml), penicillin (100 units/ml), and streptomycin (100 μg/ml). All cells were incubated at 37 °C in a humidified atmosphere of 5% CO_2._ To generate the AML12 cells that stably express Fendrr, AML12 cells were infected with the recombinant lentivirus expressing Fendrr (lv-Fendrr) or the control virus (lv-Ctrl), respectively. After lentivirus infection, the stable cell lines were selected using 0.5 mg/ml of puromycin. The stable lines were maintained in DMEM supplemented with 10% fetal bovine serum and 0.25 mg/ml of puromycin. The stable expression of Fendrr was confirmed by qRT-PCR. To collect conditioned medium, cells were washed with PBS twice and continued to incubate with serum-free culture medium for 48 h. The conditioned medium was then collected by centrifugation, and stored at −80 °C.

### Isolation of hepatocytes

Primary hepatocytes were isolated by pronase/collagenase perfusion digestion followed by subsequent density gradient centrifugation as previously described (50). The isolated primary hepatocytes were counted with a hemocytometer to determine the number and percentage of viable cells using the trypan blue method. The isolated cells with the viability above 90% were used for the experiments. To confirm the purity of isolated hepatocytes and nonhepatocytes, the expression of hepatocyte-specific alpha-fetoprotein, albumin, CYP2E1, HNF1a and HNF4a, HSC-specific Desmin, Acta2, and Syntaxin 2 (STX2), and Kupffer cell-specific CD68, Emr1, and Clec4F were examined by qRT-PCR, respectively.

### Oligonucleotides and transfection

Mouse STAT2-specific siRNA (sense: 5′- GCAGCAGAAGUCCUGCAUUTT-3′ and antisense: 5′- AAUGCAGGACUUCUGCUGCTT-3′) was designed and synthesized by GenePharma. Transfection of RNA duplexes was performed using Lipofectamine 2000 (Invitrogen). Unless otherwise indicated, the cells were transfected with the transfection mix containing 100 nM siRNA in this study.

### Wound scratch assay

Cells were grown to confluence on a 6-well plate. A linear scrape wound was made on the cell monolayer, and the wound was allowed to heal. The images were then taken under an inverted microscope (TE2000-U; Nikon) at indicated times. The distances from the edge to the middle of the scratch were determined using ImageJ software (https://imagej.nih.gov/ij/).

### Animals

Animal protocols were reviewed and approved by the Animal Care and Use Committee of Nanjing University and conformed to the Guidelines for the Care and Use of Laboratory Animals published by the National Institutes of Health. Five-week old male C57BL/6J mice (20 ± 2 g) were obtained from the Animal Center of Yangzhou University (Yangzhou, China). Animals were maintained under pathogen-limited conditions and had free access to rodent chow and water. CCl_4_-induced mouse liver fibrosis was induced according to previously described method (51). Briefly, mice were intraperitoneally injected with of 20% CCl_4_ solution in sterile mineral oil at a dose of 2.5 ml CCl_4_ per kilogram body weight twice per week for 4 weeks. The lentivirus was injected only one time *via* tail vein at 2 days before the first CCl_4_ injection (1 × 10^9^ pfu/mouse).

For BDL, we anesthetized mice with intraperitoneal injection of pentobarbital (50 mg/kg) and opened the abdominal cavity to expose the bile duct. We then ligated it twice at a distance of around 3 mm with 4-0 silk and secured them separately with three surgical knots. The sham operation group was handled in a similar manner, but without bile duct ligation. All mice were sacrificed 3 weeks later.

### Human liver tissue samples

Liver cirrhosis samples and normal control were obtained from patients undergoing surgery at Department of Hepatobiliary Surgery, the Affiliated Drum Tower Hospital of Nanjing University Medical School, Nanjing, China. Normal liver tissues were nondiseased portions of hepatectomy specimens from patients who had undergone surgery to remove liver hemangiomas. Written informed consent was obtained from each patient before collecting the sample. This study was approved by the Institutional Review Board of Nanjing University and abided by the Declaration of Helsinki principles.

### Plasmid

The Fendrr complementary DNA (cDNA) was amplified by RT-PCR with primers as follows: Forward: 5′- gcaggcttgaaggaattcggtacccgggttccggaaagc -3′ and Reverse: 5′- acaagaaagctgggttgcggccgctgatagaatttgcattttaatttatgagcaaa -3′. The PCR fragment was inserted into pMD18-T vector, sequenced and then cloned into lentiviral vector pLVX-IRES-Puro.

The STAT2 cDNA was amplified by RT-PCR with primers as follows: Forward: 5′- gagcaccaggatgacgataaa -3′ and Reverse: 5′- tgatctctgtcagtgggagt -3′. The PCR fragment was sequenced and cloned into pCI plasmid to obtain pCI-STAT2. The STAT2 mutant cDNA were then constructed by PCR using the primers as follows, mutant 1 (Forward: 5′- ttggctacctggattgaagac -3′ and Reverse: 5′- gattggagtctgagctgtagtg -3′), mutant 2 (Forward: 5′-atggcgcagtggga -3′ and Reverse: 5′- ttcacttctaaggactctgaatc -3′) and mutant 3 (Forward: 5′- gattagcgtggaggatctgaaa -3′ and Reverse: 5′- aaatggctgtggtagggtatg -3′)

### Generation of lentivirus

The Fendrr cDNA was cloned into the pLVX-IRES-Puro lentiviral vector to generate pLVX-IRES-Puro-Fendrr. The infectious virus particles were collected at 48 h after HEK 293T cells were transfected with pLVX-IRES-Puro-Fendrr, the packaging plasmid psPAX2 and the VSV-G envelope plasmid pMD2.G (Addgene) using the Lipofectamine 2000 reagent.

### Measurement of hepatic hydroxyproline content

The amount of hydroxyproline was determined with Hydroxyproline Testing Kit (Jiancheng Inc). About 100 mg wet liver samples were weighed and subjected to acid hydrolysis. The hepatic hydroxyproline levels were then biochemically measured and calculated according to the manufacturer’s instruction.

### Cytoplasmic and nuclear fractionations

The nucleus and cytoplasm were separated by the nuclear and cytoplasmic extraction kit (Beyotime). The RNA were then isolated from the pooled fractions for qRT-PCR.

### RNA FISH

LncRNA FISH was performed using a Ribo lncRNA FISH kit (RiboBio) according to the manufacturer’s protocol. The CY3-labeled FISH probes were designed and provided by RiboBio. Briefly, the liver sections were fixed with 4% paraformaldehyde after deparaffinization and rehydration. The sections were then hybridized with 20 μM CY3-labeled probe overnight.

### RNA pull-down and MS assay

RNA pull-down was performed as previously described (52). Briefly, biotin-labeled RNAs were transcribed *in vitro* with Biotin RNA Labeling Mix (Roche) and T7 RNA polymerase (Promega), treated with RNase-free DNase I (Promega) and purified with RNeasy Mini Kit (Qiagen). Three micrograms of biotinylated RNA in RNA structure buffer (10 mM Tris pH 7, 0.1 M KCl, and 10 mM MgCl2) was heated to 95 °C for 2 min, placed on ice for 3 min, and then left at room temperature (RT) for 30 min to allow proper secondary structure formation. Three micrograms of biotin-labeled RNA was then mixed with the extract (containing 1 mg proteins) in 500 μl RIP buffer and then incubated at RT for 1 h in each pull-down assay. Fifty microliters of washed streptavidin agarose beads (Invitrogen) were added to each binding reaction and further incubated at RT for another hour. Beads were washed briefly with RIP buffer five times and boiled in SDS buffer. Then, the retrieved proteins were resolved in SDS-PAGE gels. The band specific to sense Fendrr, as well as the corresponding gel in the antisense lane was then excised, respectively, and followed by MS identification.

### RNA immunoprecipitation

RNA immunoprecipitation analysis was performed as described before (52). Briefly, cells were cross-linked with 0.37% formaldehyde and lysed in 1 ml RIP lysis buffer (150 mM NaCl, 10 mM Hepes, pH 7.4, 3 mM MgCl2, 10% glycerol, 1% NP-40, 2 mM DTT, and 1 mM PMSF) supplemented with protease inhibitor and SUPERase•In RNase Inhibitor (1000 U/ml, Ambion). Cell extracts were coimmunoprecipitated using anti-STAT2 or preimmune IgG (negative control) antibody. RNA samples were extracted with TRIzol and detected by qRT-PCR.

### RNA isolation and quantitative real-time PCR

Total RNA was prepared from the cells or liver tissues by homogenization and purification using TRIzol Reagent (Invitrogen). For mRNA detection, 500 ng of total RNA were used for cDNA synthesis and real-time PCR was performed in triplicates using the SYBR PrimeScript RT-PCR Kit (Takara) in an ABI 7300 Fast Realtime PCR System (Applied Biosystems,) according to the manufacturer’s instructions. Individual gene expression was quantified using a standard curve and normalized to β-actin mRNA expression. The PCR conditions were as follows: 95 °C for 30 s, and then 40 cycles of amplification for 5 s at 95 °C and 30 s at 60 °C. Primers are listed in Table S2.

### Absolute quantification of RNA using qRT-PCR.

The absolute copy numbers of Fendrr and Albumin mRNA were measured using the standard curve method as previously described (53). Briefly, the plasmids containing Fendrr and Albumin cDNA were linearized and generated sense RNA transcript using *in vitro* T7 promoter transcription system (Promega). After digested with RNAse-free DNAse and purification, the transcripts were quantified on a spectrophotometer and converted to the number of copies using the previously described method (53). The quantified Fendrr and Albumin mRNA was used as the standard RNA. The total RNA from RIP, as well as the standard RNAs of Fendrr and Albumin, were reversely transcribed, respectively, using the reverse primer. The standard cDNAs were serially diluted in nuclease-free water. Serial dilutions from 10^6^ to 10^1^ copies were used for standard in a final volume of 20 μl. Quantitative PCR was then performed using the diluted cDNA standards or total cDNA from RIP as templates. Absolute quantification determines the actual copy numbers of target genes by relating the cycle threshold value to a standard curve. The final data were expressed as the copy number per 10 μg RNA.

### Histological examination

Excised liver tissues were fixed in 4% paraformaldehyde, embedded in paraffin and sectioned. The tissue was then stained with H&E or Sirius red. For Sirius red staining, the sections were stained for 1 h in 0.1% (w/v) sirius red (Sigma-Aldrich) in a saturated aqueous solution of picric acid (Sigma-Aldrich). After staining, the slides were rinsed twice with two changes of acidified water. After dehydration in three changes of 100% ethanol, the slides were mounted and observed. For semiquantitative analysis of liver fibrosis, at least 10 photographs of noncontiguous sections per animal were recorded. We calculated the fibrotic area relative to the total area of the field as a percentage using Image-Pro Plus 6.0 (Media Cybernetics, https://mediacy.com/products/image-pro-plus/).

### Immunofluorescence staining

Immunofluorescence staining was performed using the protocols previously established in our laboratory. Briefly, the paraffin-embedded liver tissue were sectioned and incubated with the primary antibody at 4 °C overnight according to the manufacturer’s recommendation. After the sections were washed with Tris-buffered saline, they were subsequently stained with FITC-conjugated secondary antibodies. The nuclei were counterstained with 4,6-diamidino-2-phenylindole (DAPI), and the fluorescence was visualized under Nikon confocal microscope (C2+, Nikon). The positive staining area was quantified by scanning at least 10 photographs of noncontiguous sections per animal. The results were expressed as a percentage of the total tissue area.

### Immunohistochemical staining

We performed immunohistochemistry in paraffin sections using a microwave-based antigen retrieval technique. Following deparaffinization, sections were rehydrated and subjected to antigen retrieval by microwaving in 0.01 M sodium citrate (pH 6.0) for 10 min. Sections were incubated at 4 °C overnight with primary antibody and then incubated with horseradish peroxidase-conjugated IgG (1:500) according to the manufacturer’s instructions. The proteins *in situ* were visualized with 3,3-diaminobenzidine and examined under confocal microscopy. The staining intensity was quantified using Image-Pro Plus 6.0 by scanning 10 nonoverlapping fields in each liver section.

### Western blot

Cell pellets or liver tissues were lysed in lysis buffer (20 mM Tris–HCl, pH 7.5, 150 mM NaCl, 1% Triton X-100, sodium pyrophosphate, β-glycerophosphate, EDTA, Na_3_VO_4_ and leupeptin) containing 1 mM PMSF. After mixing with 2 × Laemmli buffer and heat denaturation at 100 °C for 5 min, equal amounts of lysate (20 μl) were separated by SDS-PAGE and then transferred onto polyvinylidene fluoride membranes. The membranes were probed by antibodies at appropriate dilutions for 1 h. After washing with phosphate buffered saline with Tween-20, the membrane was then probed with horseradish peroxidase-conjugated anti-rabbit or anti-goat IgG for 1 h at RT. The protein bands were visualized by fluorography using an enhanced chemiluminescence system (Cell Signaling Technology).

### Serum aminotransferase activity measurement

The blood samples were allowed to clot, and then centrifuged at 3000*g* for 10 min to collect serum for aminotransferase activity analysis. ALT was measured using commercial kits (Jiancheng) and expressed as U/L.

### ELISA assay

Quantification of IL-6 production was performed with a commercial ELISA kit (Multisciences Biotech) according to the manufacturer's instructions.

### Statistical analysis

Results are expressed as the means ± SD of at least three independent experiments. The data were analyzed for normal distribution. Differences between multiple groups were checked using one-way ANOVA with *post hoc* Bonferroni correction. Differences between two groups were analyzed by a two-tailed unpaired Student’s *t* test. A value of *p* < 0.05 was considered statistically significant, and *p* < 0.01, *p* < 0.001 and *p* < 0.0001 indicated strongly significant difference. Pearson’s correlation analysis was performed to determine the correlation between two variables.

## Data availability

The RNA-seq data have been deposited in the Gene Expression Omnibus (GEO) under the accession code GSE243644 (https://www.ncbi.nlm.nih.gov/geo/query/acc.cgi?acc=GSE243644). The data that support the findings of this study are available from the corresponding author upon reasonable request.

## Supporting information

This article contains supporting information.

## Conflict of interest

The authors declare that they have no conflicts of interest with the contents of this article.

## References

[1] Friedman S.L. (2010). Evolving challenges in hepatic fibrosis. Nat. Rev. Gastroenterol. Hepatol..

[2] Bataller R., Brenner D.A. (2005). Liver fibrosis. J. Clin. Invest..

[3] Zhang D.Y., Friedman S.L. (2012). Fibrosis-dependent mechanisms of hepatocarcinogenesis. Hepatology.

[4] Lee Y.A., Wallace M.C., Friedman S.L. (2015). Pathobiology of liver fibrosis: a translational success story. Gut.

[5] Ellis E.L., Mann D.A. (2012). Clinical evidence for the regression of liver fibrosis. J. Hepatol..

[6] Mederacke I., Hsu C.C., Troeger J.S., Huebener P., Mu X., Dapito D.H. (2013). Fate tracing reveals hepatic stellate cells as dominant contributors to liver fibrosis independent of its aetiology. Nat. Commun..

[7] Luedde T., Kaplowitz N., Schwabe R.F. (2014). Cell death and cell death responses in liver disease: mechanisms and clinical relevance. Gastroenterology.

[8] Xiang D.M., Sun W., Ning B.F., Zhou T.F., Li X.F., Zhong W. (2018). The HLF/IL-6/STAT3 feedforward circuit drives hepatic stellate cell activation to promote liver fibrosis. Gut.

[9] Zhang K., Shi Z.M., Chang Y.N., Hu Z.M., Qi H.X., Hong W. (2014). The ways of action of long non-coding RNAs in cytoplasm and nucleus. Gene.

[10] Yu F., Zheng J., Mao Y., Dong P., Lu Z., Li G. (2015). Long non-coding RNA growth arrest-specific transcript 5 (GAS5) inhibits liver fibrogenesis through a mechanism of competing endogenous RNA. J. Biol. Chem..

[11] Song Y., Liu C., Liu X., Trottier J., Beaudoin M., Zhang L. (2017). H19 promotes cholestatic liver fibrosis by preventing ZEB1-mediated inhibition of epithelial cell adhesion molecule. Hepatology.

[12] Wang C., Deng J., Deng H., Kang Z., Huang Z., Ding Z. (2020). A novel Sox9/lncRNA H19 axis contributes to hepatocyte death and liver fibrosis. Toxicol. Sci..

[13] Sauvageau M., Goff L.A., Lodato S., Bonev B., Groff A.F., Gerhardinger C. (2013). Multiple knockout mouse models reveal lincRNAs are required for life and brain development. Elife.

[14] Grote P., Wittler L., Hendrix D., Koch F., Währisch S., Beisaw A. (2013). The tissue specific lncRNA Fendrr is an essential regulator of heart and body wall development in the mouse. Dev. Cell.

[15] Lai K.M., Gong G., Atanasio A., Rojas J., Quispe J., Posca J. (2015). Diverse phenotypes and specific transcription patterns in twenty mouse lines with ablated lincRNAs. PLoS One.

[16] Fagerberg L., Hallström B.M., Oksvold P., Kampf C., Djureinovic D., Odeberg J. (2014). Analysis of the human tissue-specific expression by genome-wide integration of transcriptomics and antibody-based proteomics. Mol. Cell. Proteom..

[17] Yu Z., Zhao H., Feng X., Li H., Qiu C., Yi X. (2019). Long non-coding RNA FENDRR acts as a miR-423-5p sponge to suppress the Treg-mediated immune escape of hepatocellular carcinoma cells. Mol. Ther. Nucleic Acids.

[18] Xu T.P., Huang M.D., Xia R., Liu X.X., Sun M., Yin L. (2014). Decreased expression of the long non-coding RNA FENDRR is associated with poor prognosis in gastric cancer and FENDRR regulates gastric cancer cell metastasis by affecting fibronectin1 expression. J. Hematol. Oncol..

[19] Huang C., Liang Y., Zeng X., Yang X., Xu D., Gou X. (2020). Long Noncoding RNA FENDRR exhibits antifibrotic activity in pulmonary fibrosis. Am. J. Respir. Cell Mol. Biol..

[20] Gong L., Zhu L., Yang T. (2020). Fendrr involves in the pathogenesis of cardiac fibrosis via regulating miR-106b/SMAD3 axis. Biochem. Biophys. Res. Commun..

[21] Zhang F., Ni H., Li X., Liu H., Xi T., Zheng L. (2019). LncRNA FENDRR attenuates adriamycin resistance via suppressing MDR1 expression through sponging HuR and miR-184 in chronic myelogenous leukaemia cells. FEBS Lett..

[22] Khalil A.M., Guttman M., Huarte M., Garber M., Raj A., Rivea Morales D. (2009). Many human large intergenic noncoding RNAs associate with chromatin-modifying complexes and affect gene expression. Proc. Natl. Acad. Sci. U. S. A..

[23] Nan J., Wang Y., Yang J., Stark G.R. (2018). IRF9 and unphosphorylated STAT2 cooperate with NF-κB to drive IL6 expression. Proc. Natl. Acad. Sci. U. S. A..

[24] Blaszczyk K., Nowicka H., Kostyrko K., Antonczyk A., Wesoly J., Bluyssen H.A.R. (2016). The unique role of STAT2 in constitutive and IFN-induced transcription and antiviral responses. Cytokine Growth Factor Rev..

[25] Granzow M., Schierwagen R., Klein S., Kowallick B., Huss S., Linhart M. (2014). Angiotensin-II type 1 receptor-mediated Janus kinase 2 activation induces liver fibrosis. Hepatology.

[26] Jiang J.X., Mikami K., Venugopal S., Li Y., Török N.J. (2009). Apoptotic body engulfment by hepatic stellate cells promotes their survival by the JAK/STAT and akt/NF-kappaB-dependent pathways. J. Hepatol..

[27] Platanias L.C. (2005). Mechanisms of type-I- and type-II-interferon-mediated signalling. Nat. Rev. Immunol..

[28] Alazawi W., Heath H., Waters J.A., Woodfin A., O'Brien A.J., Scarzello A.J. (2013). Stat2 loss leads to cytokine-independent, cell-mediated lethality in LPS-induced sepsis. Proc. Natl. Acad. Sci. U. S. A..

[29] Ho J., Pelzel C., Begitt A., Mee M., Elsheikha H.M., Scott D.J. (2016). STAT2 is a pervasive cytokine regulator due to its inhibition of stat1 in multiple signaling pathways. PLoS Biol..

[30] Levy D.E., Darnell J.E. (2002). Stats: transcriptional control and biological impact. Nat. Rev. Mol. Cell Biol..

[31] Martinez-Moczygemba M., Gutch M.J., French D.L., Reich N.C. (1997). Distinct STAT structure promotes interaction of STAT2 with the p48 subunit of the interferon-alpha-stimulated transcription factor ISGF3. J. Biol. Chem..

[32] Banninger G., Reich N.C. (2004). STAT2 nuclear trafficking. J. Biol. Chem..

[33] Kraus T.A., Lau J.F., Parisien J.P., Horvath C.M. (2003). A hybrid IRF9-STAT2 protein recapitulates interferon-stimulated gene expression and antiviral response. J. Biol. Chem..

[34] Paulson M., Pisharody S., Pan L., Guadagno S., Mui A.L., Levy D.E. (1999). Stat protein transactivation domains recruit p300/CBP through widely divergent sequences. J. Biol. Chem..

[35] Lau J.F., Parisien J.P., Horvath C.M. (2000). Interferon regulatory factor subcellular localization is determined by a bipartite nuclear localization signal in the DNA-binding domain and interaction with cytoplasmic retention factors. Proc. Natl. Acad. Sci. U. S. A..

[36] Tamura T., Yanai H., Savitsky D., Taniguchi T. (2008). The IRF family transcription factors in immunity and oncogenesis. Annu. Rev. Immunol..

[37] Melen K., Kinnunen L., Julkunen I. (2001). Arginine/lysine-rich structural element is involved in interferon-induced nuclear import of STATs. J. Biol. Chem..

[38] Begitt A., Krause S., Cavey J.R., Vinkemeier D.E., Vinkemeier U.A. (2023). Family-wide assessment of latent STAT transcription factor interactions reveals divergent dimer repertoires. J. Biol. Chem..

[39] Lackmann M., Harpur A.G., Oates A.C., Mann R.J., Gabriel A., Meutermans W. (1998). Biomolecular interaction analysis of IFN gamma-induced signaling events in whole-cell lysates: prevalence of latent STAT1 in high-molecular weight complexes. Growth Factors.

[40] Ndubuisi M.I., Guo G.G., Fried V.A., Etlinger J.D., Sehgal P.B. (1999). Cellular physiology of STAT3: where's the cytoplasmic monomer?. J. Biol. Chem..

[41] Zhang M.Y., Zhang Z.L., Cui H.X., Wang R.K., Fu L. (2018). Long non-coding RNA FENDRR inhibits NSCLC cell growth and aggressiveness by sponging miR-761. Eur. Rev. Med. Pharmacol. Sci..

[42] Senavirathna L.K., Huang C., Pushparaj S., Xu D., Liu L. (2020). Hypoxia and transforming growth factor β1 regulation of long non-coding RNA transcriptomes in human pulmonary fibroblasts. Physiol. Rep..

[43] Yanger K., Knigin D., Zong Y., Maggs L., Gu G., Akiyama H. (2014). Adult hepatocytes are generated by self-duplication rather than stem cell differentiation. Cell Stem Cell.

[44] Schaub J.R., Malato Y., Gormond C., Willenbring H. (2014). Evidence against a stem cell origin of new hepatocytes in a common mouse model of chronic liver injury. Cell Rep..

[45] Szafranski P., Gambin T., Karolak J.A., Popek E., Stankiewicz P. (2021). Lung-specific distant enhancer cis regulates expression of FOXF1 and lncRNA FENDRR. Hum. Mutat..

[46] Ren X., Ustiyan V., Pradhan A., Cai Y., Havrilak J.A., Bolte C.S. (2014). FOXF1 transcription factor is required for formation of embryonic vasculature by regulating VEGF signaling in endothelial cells. Circ. Res..

[47] Flood H.M., Bolte C., Dasgupta N., Sharma A., Zhang Y., Gandhi C.R. (2019). The Forkhead box F1 transcription factor inhibits collagen deposition and accumulation of myofibroblasts during liver fibrosis. Biol. Open.

[48] Abshagen K., Rotberg T., Genz B., Vollmar B. (2017). No significant impact of Foxf1 siRNA treatment in acute and chronic CCl4 liver injury. Exp. Biol. Med. (Maywood)..

[49] Abshagen K., Brensel M., Genz B., Roth K., Thomas M., Fehring V. (2015). Foxf1 siRNA delivery to hepatic stellate cells by DBTC lipoplex formulations ameliorates fibrosis in livers of bile duct ligated mice. Curr. Gene Ther..

[50] Weiskirchen R., Gressner A.M. (2005). Isolation and culture of hepatic stellate cells. Methods Mol. Med..

[51] Tu X., Zhang H., Zhang J., Zhao S., Zheng X., Zhang Z. (2014). MicroRNA-101 suppresses liver fibrosis by targeting the TGFβ signalling pathway. J. Pathol..

[52] Chen C., He W., Huang J., Wang B., Li H., Cai Q. (2018). LNMAT1 promotes lymphatic metastasis of bladder cancer via CCL2 dependent macrophage recruitment. Nat. Commun..

[53] Wang K., Liu C.Y., Zhou L.Y., Wang J.X., Wang M., Zhao B. (2015). APF lncRNA regulates autophagy and myocardial infarction by targeting miR-188-3p. Nat. Commun..

