# Supplementary material for: The increase of long noncoding RNA Fendrr in hepatocytes contributes to liver fibrosis by promoting IL-6 production
Source: J Biol Chem. 2024 May 16;300(6):107376. doi: 10.1016/j.jbc.2024.107376 (PMC11190708; doi:10.1016/j.jbc.2024.107376)
Supplement: Supplementary Table and Figures [file mmc1.pdf]

The increase of long noncoding RNA Fendrr in hepatocytes contributes to liver fibrosis by promoting IL-6 production

Zhiqian Kang[1], Chenqi Wang[1], Fang Shao[1], Hao Deng[1], Yanyan Sun[1,2], Zhengrong Ren[1], Wei Zhang[1], Zhi Ding[1], Junfeng Zhang[1,*], Yuhui Zang[1,*]

[1]*State Key Laboratory of Pharmaceutical Biotechnology, School of Life Sciences, Nanjing University, Nanjing 210093, PR China*

[2] *State Key Laboratory for Organic Electronics and Information Displays (SKLOEID) & Jiangsu Key Laboratory for Biosensors, Institute of Advanced Materials (IAM), Jiangsu National Synergistic Innovation Center for Advanced Materials (SICAM), School of Chemistry and Life Sciences, Nanjing University of Posts and Telecommunications, Nanjing 210023, China.*

Supplementary Table S1 Identification of Fendrr binding proteins by MS

The table was submitted in its native format as separate file.

Supplementary Table S2 qRT-PCR primers used in this study

Gene name	Primer name	Primer Sequence
Mouse Fendrr	Forward (5′- 3′)	ctgcccgtgtggttataatg
	Reverse (5′- 3′)	tgactctcaagtgggtgctg
Human Fendrr	Forward (5′- 3′)	agtgcactgtgtgctcttag
	Reverse (5′- 3′)	gaggatctgtggttgggtattt
Mouse Col1a1	Forward (5′- 3′)	gaaacccgaggtatgcttga
	Reverse (5′- 3′)	gttgggacagtccagttctt
Mouse Tgfb1	Forward (5′- 3′)	cgaagcggactactatgctaaa
	Reverse (5′- 3′)	tcccgaatgtctgacgtattg
Mouse/Rat Acta2	Forward (5′- 3′)	ttgctgacaggatgcagaagg
	Reverse (5′- 3′)	ctgatccacatctgctggaag
Mouse/Rat β-actin	Forward (5′- 3′)	gacctctatgccaacacagtgc
	Reverse (5′- 3′)	gtactcctgcttgctgatccac
Mouse Timp1	Forward (5′- 3′)	ggcatctggcatcctcttgt
	Reverse (5′- 3′)	tggtctcgttgatttctgggg
Mouse Pdgfa	Forward (5′- 3′)	aggctgctgtaagttgggac
	Reverse (5′- 3′)	aaaggggcagaggaacacag
Mouse STAT2	Forward (5′- 3′)	gacactctccctgttgtgattat
	Reverse (5′- 3′)	actgctggttcttgggattt
Mouse STAT2-Mut1	Forward (5′- 3′)	gagatatgttcgggcagggt
	Reverse (5′- 3′)	agagtctggggcatgcag
Mouse IL-6	Forward (5′- 3′)	gataagctggagtcacagaagg
	Reverse (5′- 3′)	ttgccgagtagatctcaaagtg
Mouse Albumin	Forward (5′- 3′)	cgccaactgtgacaaatccc
	Reverse (5′- 3′)	acagcagtcagccagttcac
Mouse AFP	Forward (5′- 3′)	acggagaagaatgtgcttagca
	Reverse (5′- 3′)	tttctaaacacccatcgccaga
Mouse CYP2E1	Forward (5′- 3′)	gggaatggggaaacagggtaat
	Reverse (5′- 3′)	cagccaatcagaaaggtagggt
Mouse Desmin	Forward (5′- 3′)	aggagatccgacacctaaagga
	Reverse (5′- 3′)	acatccaaggccatcttcacat
Mouse CD68	Forward (5′- 3′)	ctaggaccgcttatagcccaag
	Reverse (5′- 3′)	ttctgtggctgtaggtgtcatc
Mouse Clec4F	Forward (5′- 3′)	tttccagggatctcctttctgc
	Reverse (5′- 3′)	gctctttgcagtttgagggaag

Mouse Emr1	Forward (5′- 3′)	tgtctgaagattctcaaaacatgga
	Reverse (5′- 3′)	agtcttgtgtacgatgcatggt
Mouse HNF1a	Forward (5′- 3′)	gcccggtccgttgact
	Reverse (5′- 3′)	cttagaaaccatggctccgctg
Mouse HNF4a	Forward (5′- 3′)	tgaccatgggcaatgacacg
	Reverse (5′- 3′)	ctcgaggctccgtagtgttt
Mouse STX2	Forward (5′- 3′)	cggccaccggagagtgtg
	Reverse (5′- 3′)	cagcagtgtctccatcgtcg
Human IL-6	Forward (5′- 3′)	cactcacctcttcagaacgaat
	Reverse (5′- 3′)	gctgctttcacacatgttactc
Human Col1a1	Forward (5′- 3′)	ctaaaggcgaacctggtgat
	Reverse (5′- 3′)	tccaggagcaccaacattac
Human Acta-2	Forward (5′- 3′)	gatggtgggaatgggacaaa
	Reverse (5′- 3′)	gccatgttctatcgggtacttc
Human β-actin	Forward (5′- 3′)	gacctgtacgccaacacagtgc
	Reverse (5′- 3′)	atactcctgcttgctgatccac

Supplementary figure

Supplementary Figure S1

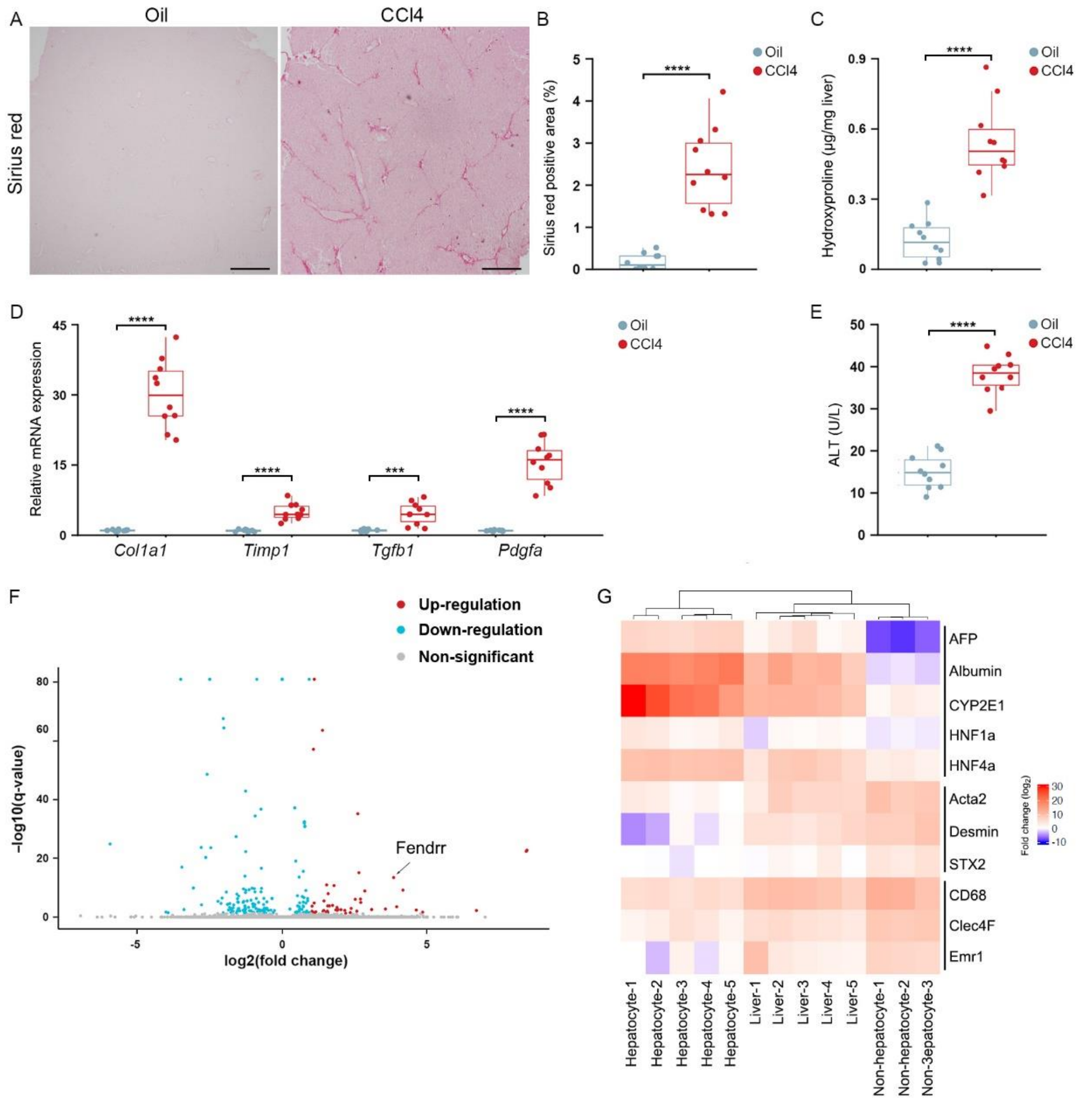

Supplementary Figure S1. CCl_4 induces liver fibrosis and the upregulation of Fendrr in mice. (A) Representative sirius red staining images of CCl_4-induced fibrotic liver. Scale bar, 100 μm. (B) Quantification of the sirius red positive area. (C) Quantitative evaluation of hepatic hydroxyproline. The hydroxyproline contents are expressed as μg/mg wet liver weight. (D) Hepatic *Tgfb1*, *Pdgf*, *Col1a1* and *Timp1* mRNA expression were examined by qRT-PCR. The results are shown as fold change compared with oil-injected mice. (E) Assessment of serum ALT levels. (F) Differential lncRNA expression in the hepatocytes isolated from CCl_4-induced fibrotic liver. The arrow points

to Fendrr. Data are the mean±SD of three independent experiments. (G) Purity assay of hepatocytes and non-hepatocytes isolated from mouse liver. The expression levels of hepatocyte-specific alpha-fetoprotein (AFP), Albumin, CYP2E1, HNF1a, HNF4a, HSC-specific Desmin, Acta2, Syntaxin 2 (STX2), and kupffer cell-specific CD68, Emr1, Clec4F, were examined by qRT-PCR in the isolated hepatocytes, non-hepatocytes, and liver tissues respectively. The heatmap represents the listed genes differentially expressed in the isolated hepatocytes and non-hepatocytes. ***$P < 0.001$, ****$P < 0.0001$.

Supplementary Figure S2

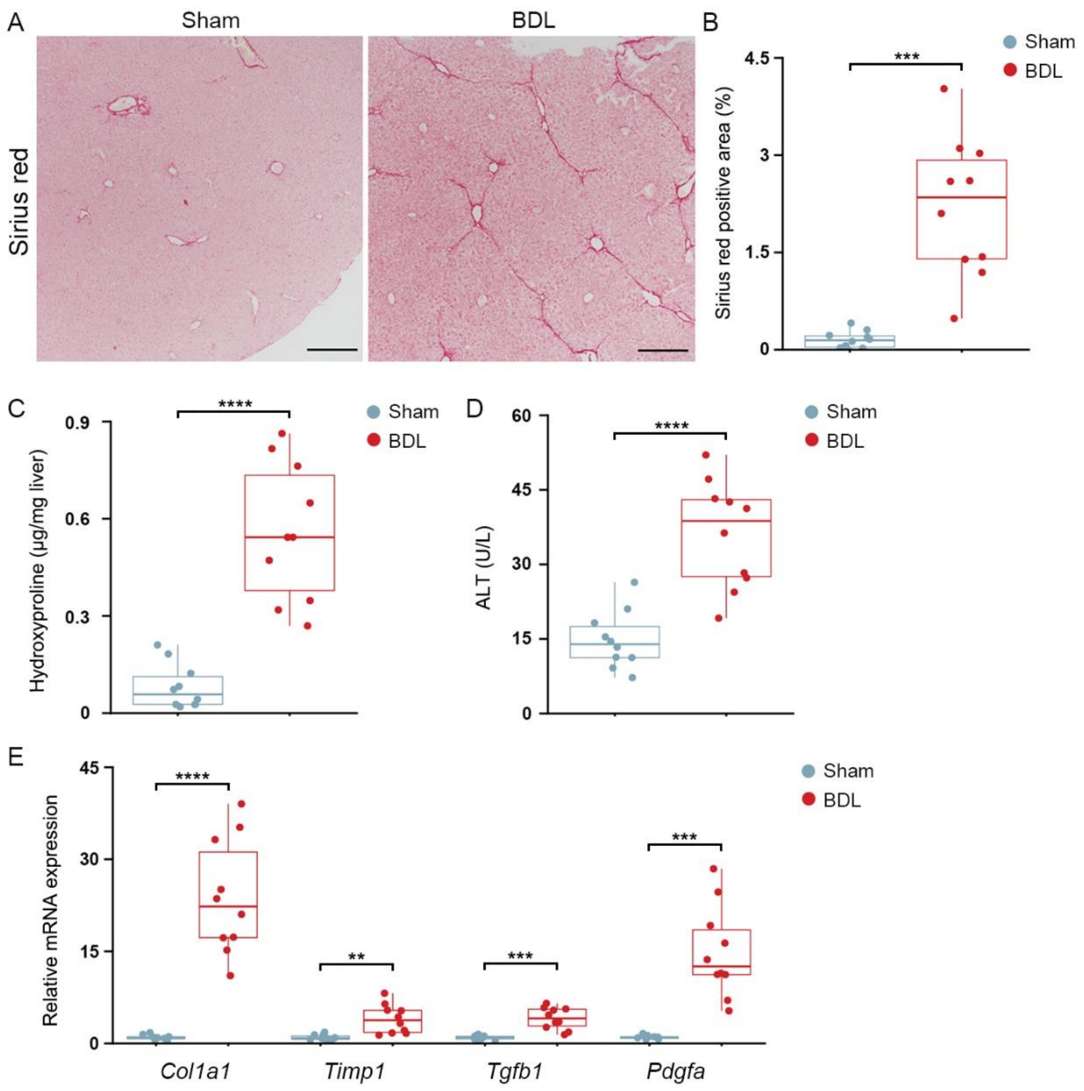

Supplementary Figure S2. BDL induces liver fibrosis in mice. (A) Representative sirius red staining images of BDL-induced fibrotic liver. Scale bar, 100 μm. (B) Quantification of the sirius red positive area. (C) Quantitative evaluation of hepatic hydroxyproline. (D) Assessment of serum ALT levels. (E) Hepatic *Tgfb1*, *Pdgf*, *Col1a1* and *Timp1* mRNA expression were examined by qRT-PCR. The results are shown as fold change compared with sham operation mice. Data are the mean±SD of three independent experiments. ***P* < 0.01, ****P* < 0.001, *****P* < 0.0001.

Supplementary Figure S3

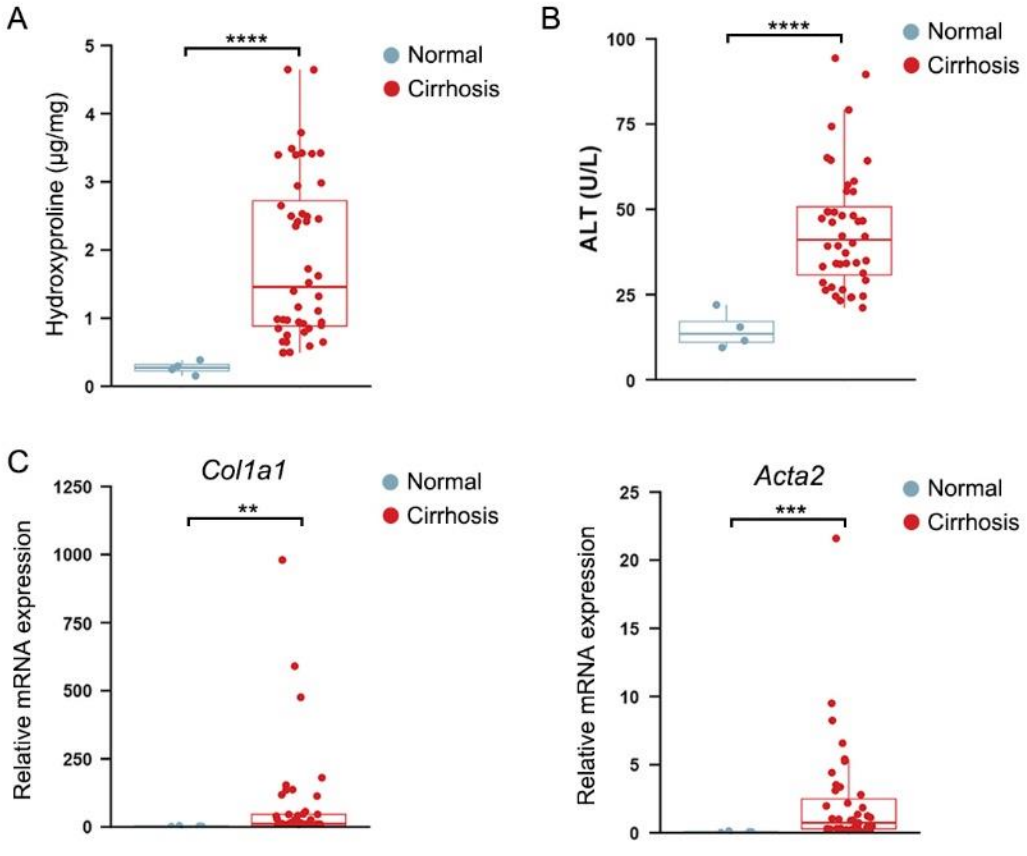

Supplementary Figure S3. Characterization of the specimens from patients with liver cirrhosis (n=41). Non-diseased liver tissue from patients undergoing hepatectomy of hemangiomas was used as normal control (n=4). (A) Quantitative evaluation of hepatic hydroxyproline. (B) Assessment of serum ALT. (C) Hepatic *Col1a1* and *Acta2* mRNA were examined by qRT-PCR. The results are shown as fold change compared with normal control. Data are the mean±SD of three independent experiments. **$P < 0.01$, ***$P < 0.001$, ****$P < 0.0001$.

Supplementary Figure S4

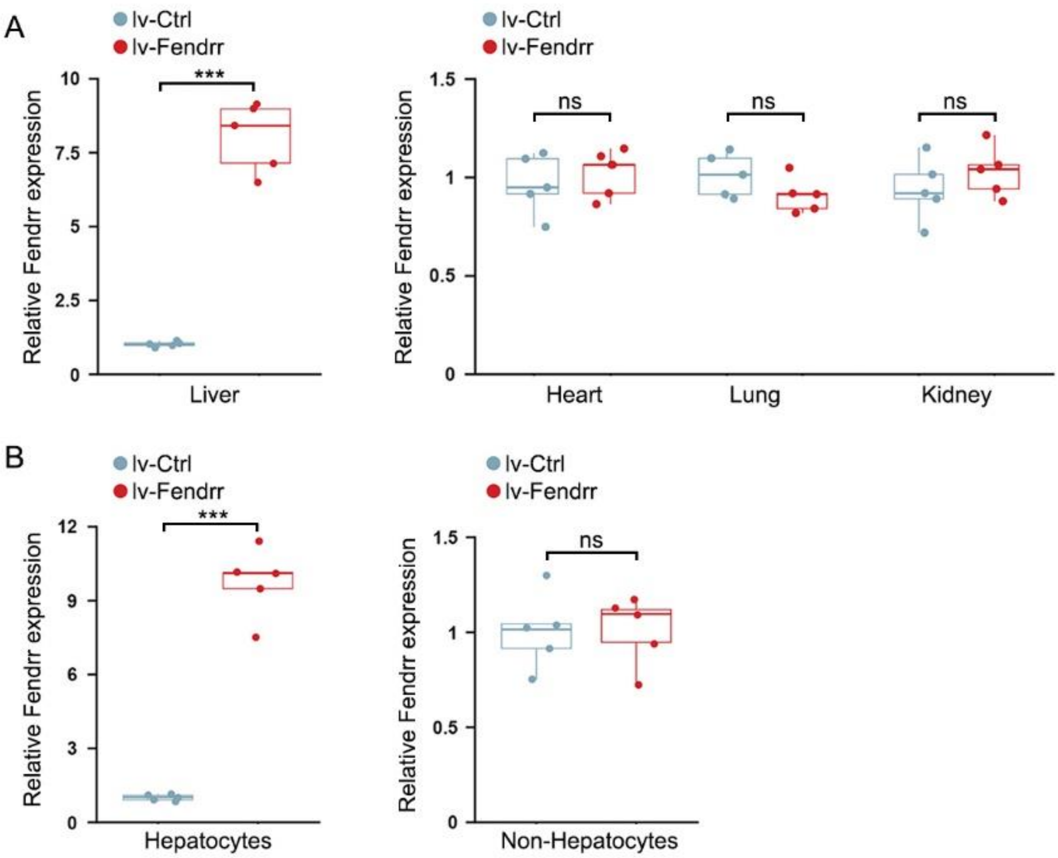

Supplementary Figure S4. The recombinant lentivirus lv-Fendrr is capable of expressing Fendrr in hepatocytes *in vivo*. lv-Fendrr or lv-Ctrl was injected into mice via tail vein and the mice was sacrificed at 4 weeks later. (A) Fendrr expression was examined by qRT-PCR in liver, heart, kidney and lung. (B) Injection of lv-Fendrr led to a significant increase of Fendrr in the hepatocytes, but not non-hepatocytes. Hepatocytes were isolated from the lentivirus-injected mice. The results are shown as fold change compared with mice injected with lv-Ctrl. n=5 for each group. Data are the mean±SD of three independent experiments. ***$P < 0.001$, and ns, no significant.

Supplementary Figure S5

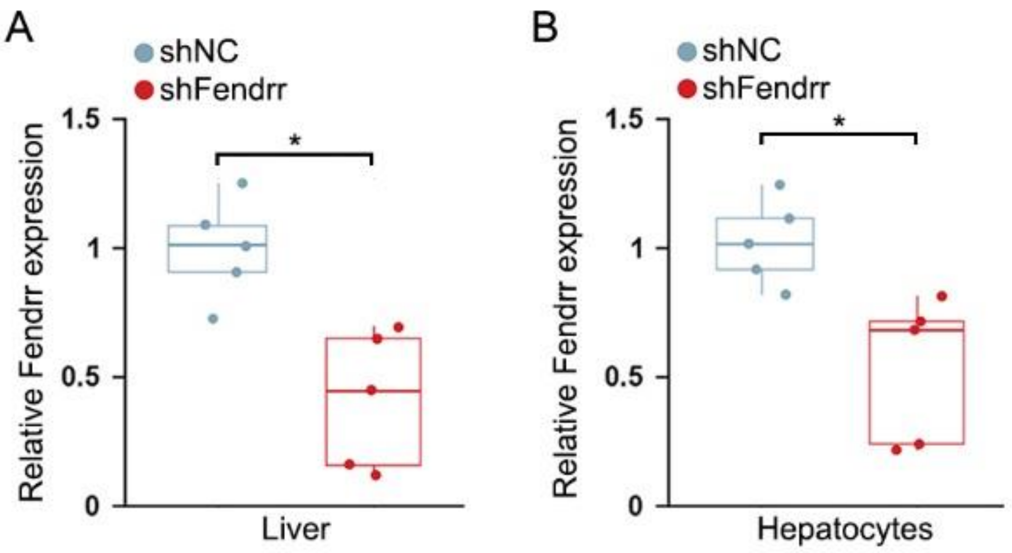

Supplementary Figure S5 Fendrr shRNA significantly reduced the expression of Fendrr in hepatocytes *in vivo*. The lentivirus overexpressing shFendrr was injected in to mice via tail vein and the mice was sacrificed at 4 weeks later. Fendrr expression was examined by qRT-PCR in the liver (A) and the isolated hepatocytes (B). The results are shown as fold change compared with mice injected with control lentivirus (shNC). n=5 for each group. Data are the mean±SD of three independent experiments. *$P < 0.05$.

Supplementary Figure S6

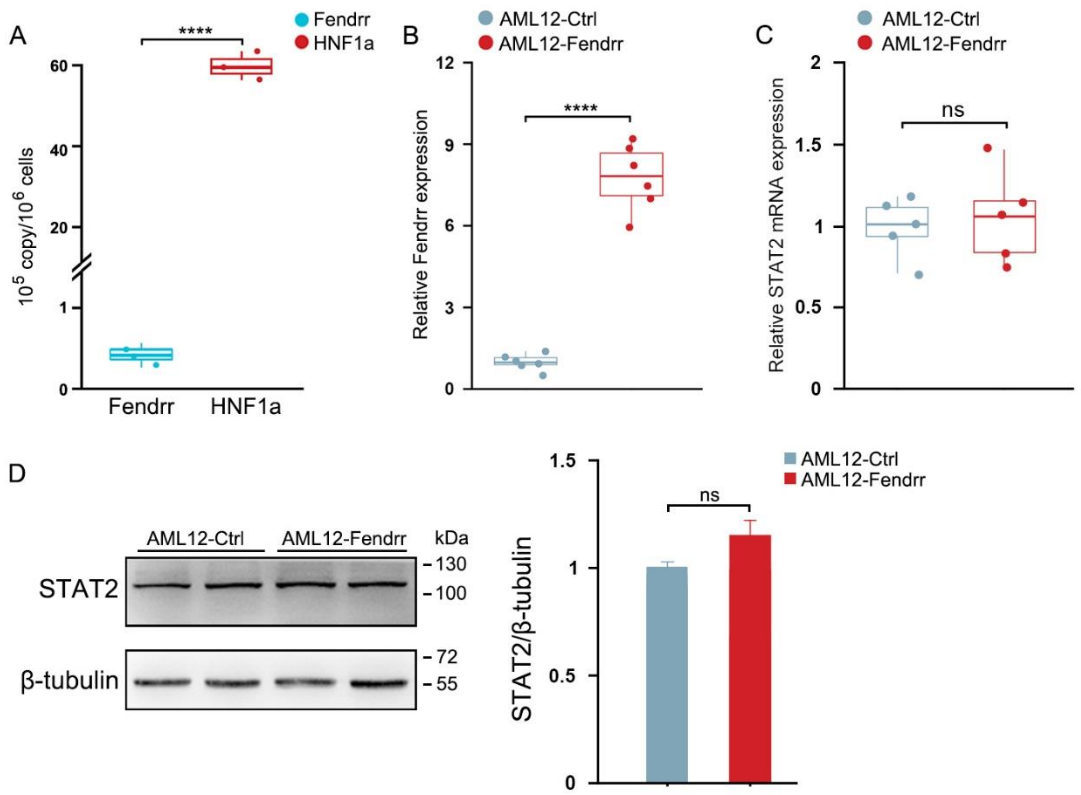

Supplementary Figure S6. Fendrr exhibits no effect on STAT2 expression. (A) Detection of Fendrr in AML12 cells. The copy number of Fendrr, as well as that of *HNF1a* mRNA, in $1×10^6$ AML12 cell was determined by absolute quantification assay. The results were shown as the copy per 10^6 cells. (B) qRT-PCR measurement of Fendrr in the AML12-Fendrr sublines. The results are shown as fold change compared with AML12-Ctrl cells. (C and D) STAT2 expression was assayed by qRT-PCR (C) and western blot (D). The qRT-PCR result are shown as fold change compared with AML12-Ctrl cells. Data are the mean±SD of three independent experiments. ****P < 0.0001, and ns, no significant.

Supplementary Figure S7

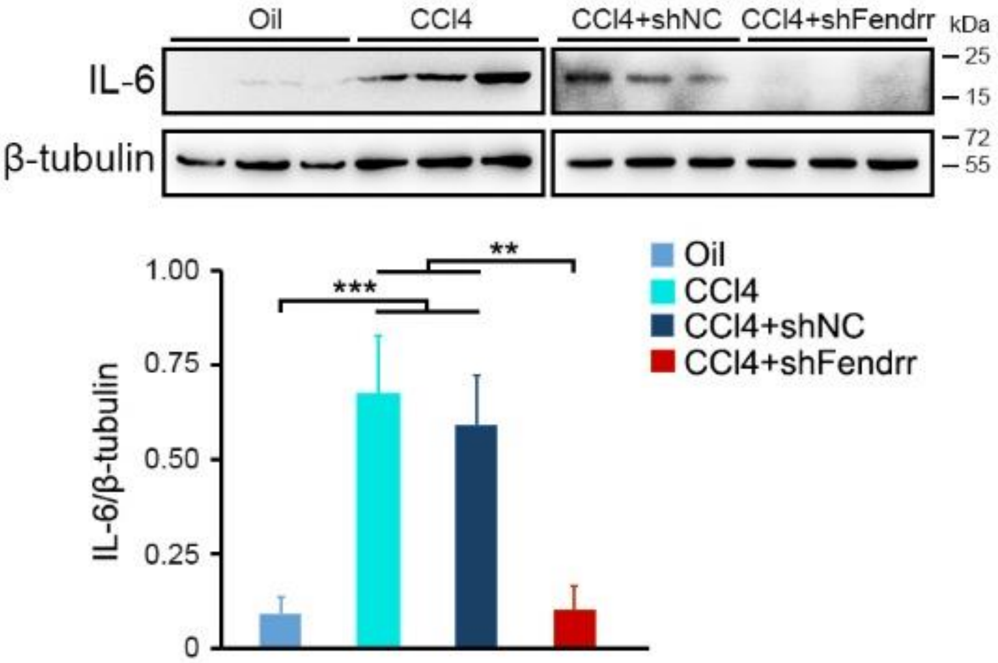

Supplementary Figure S7. Knockdown of Fendrr suppressed the upregulation of IL-6 in the CCl_4-induced fibrotic liver. Mice were injected with oil (Oil), CCl_4 (CCl4), CCl_4 in combination with injection of control lentivirus (CCl4+shNC) and CCl_4 in combination with injection of lentivirus capable of expressing Fendrr shRNA (CCl4+shFendrr). The lentivirus was injected once at two days before the first CCl_4 injection. IL-6 was detected by western blot (top). Relative protein level was calculated respectively by band intensity against β-tubulin (bottom).

Supplementary Figure S8

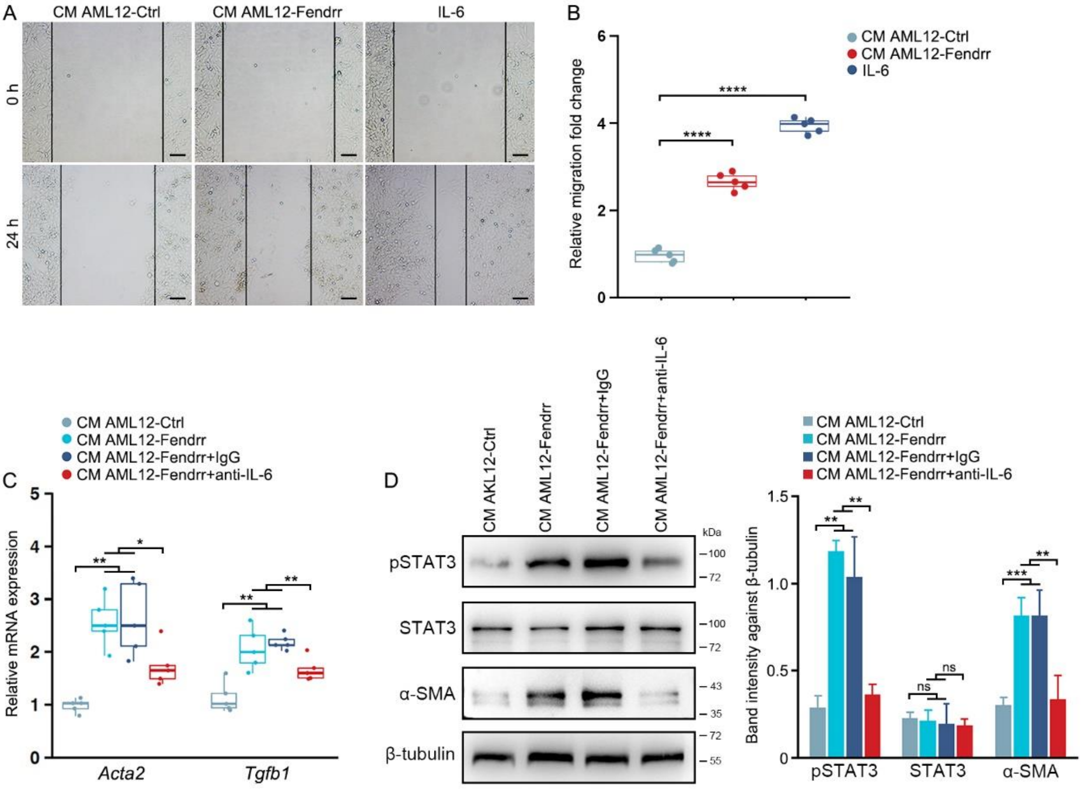

Supplementary Figure S8. (A and B) The CM from Fendrr overexpressing hepatocytes promotes the migration of HSC. HSC-T6 cells were incubated with IL-6 at a final concentration of 10 ng/ml or the CM from AML12-Fendrr subline for 24 hours. The HSC cell migration was measured by wound scratch assay at indicated times (A). The migration rate was calculated as the ratio to the relative migrating width, and the results are shown as fold change relative to the cell treated with the CM form AML12-Ctrl cells (B). Scale bar, 50 μm. (C and D) The neutralizing antibody against IL-6 suppresses the profibrotic potentials of the CM from AML12-Fendrr cells. HSC-T6 cells were incubated with the CM in the presence of neutralizing antibody or IgG for 48 hours. TGF-β1 and α-SMA expressions were assayed by qRT-PCR (C). STAT3, pSTAT3 and α-SMA were detected by western blot. Relative protein level was calculated respectively by band intensity against β-tubulin (D). Data are the mean±SD of five independent experiments. ****$P < 0.0001$.

Supplementary Figure S9

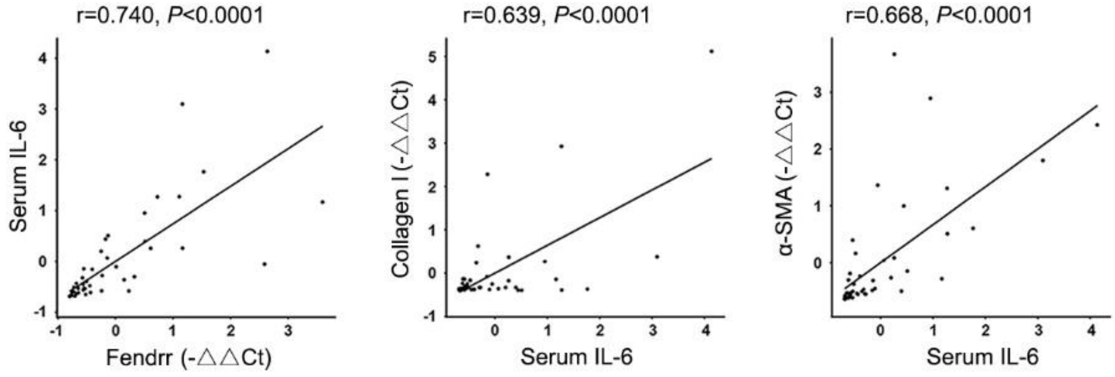

Supplementary Figure S9. Serum IL-6 is positively correlated with sirius red-stained area, and the mRNA expression of collagen I and α-SMA in the specimens from liver cirrhosis patients respectively. The expressions of collagen I and α-SMA mRNA were determined by qRT-PCR. Serum IL-6 concentration was assayed by ELISA. Data points represent measurements of individual patients (n=41). The Pearson correlation coefficient (r) is shown.

Supplementary Figure S10

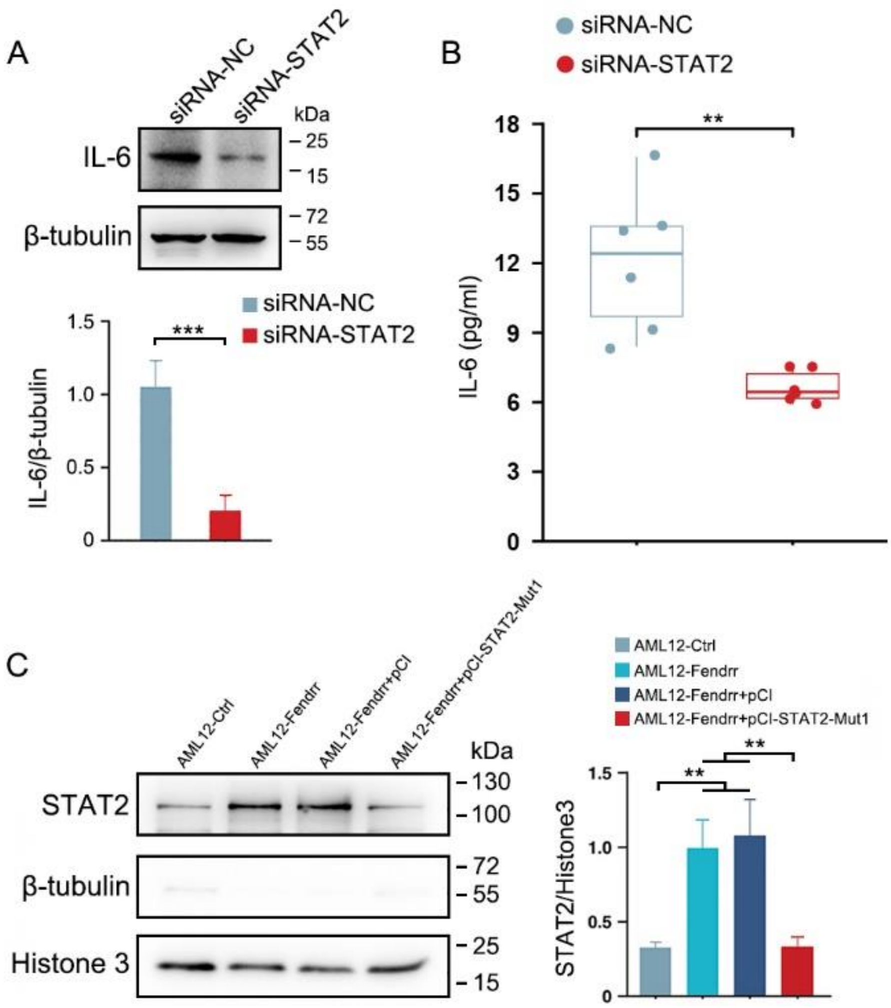

Supplementary Figure S10. Knockdown of STAT2 reduces IL-6 expression in hepatocyte. AML12-Fendrr cells were transferred with STAT2 specific (siRNA-STAT2) or scramble control (siRNA-NC) and cultured for 48 hours. (A) IL-6 were detected by western blot (top). Relative protein level was calculated respectively by band intensity against β-tubulin (bottom). (B) IL-6 product in the culture medium was assayed by ELISA. (C) STAT2-Mut1 blockade the Fendrr-mediated enrichment of STAT2 in the nuclei of hepatocytes. AML12-Fendrr cells were transferred with pCI or pCI-STAT2-Mut1 plasmid and cultured for 48 hours. Nuclear fractionation and western blot assay of STAT2 in the nucleus (left). Relative protein level was calculated respectively by band intensity against Histone 3 (right). Data are the mean±SD of at least three independent experiments. $^{**}P < 0.01$.

Supplementary Figure S11

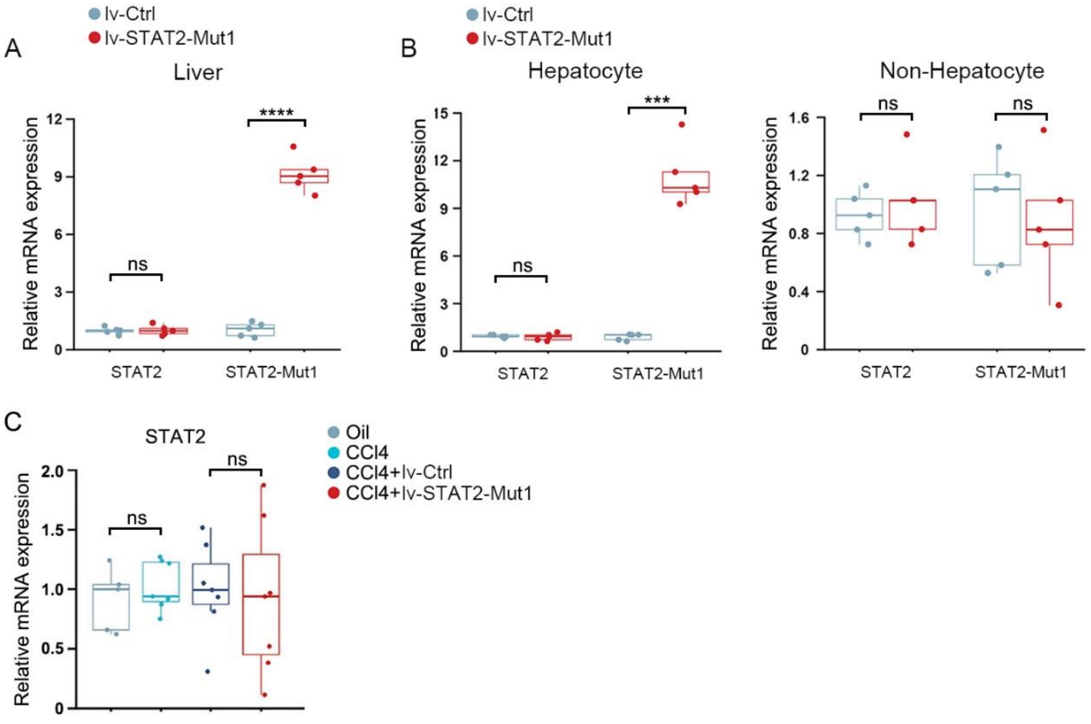

Supplementary Figure S11. (A and B) The recombinant lentivirus lv-STAT2-Mut1 is capable of expressing STAT2-Mut1 in hepatocytes *in vivo*. lv-STAT2-Mut1 or lv-Ctrl was injected into mice via tail vein and the mice was sacrificed at 4 weeks later. (A) STAT2 and STAT2-Mut1 expression was examined by qRT-PCR in liver. (B) Injection of lv-STAT2-Mut1 led to a significant ectopic expression of STAT2-Mut1 in the hepatocytes, but not in non-hepatocytes. Hepatocytes were isolated from the lentivirus-injected mice. The results are shown as fold change compared with mice injected with lv-Ctrl. n=5 for each group. (C) The hepatic STAT2 expression did not altered after CCl_4 treatment and lv-STAT2-Mut1 injection. Mice were injected with oil (Oil, n=5), CCl_4 (CCl_4, n=7), CCl_4 in combination with injection of control lentivirus (CCl4+lv-Ctrl, n=7), and CCl_4 in combination with injection of lv-STAT2-Mut1 (CCl4+lv-STAT2-Mut1, n=7). Hepatic STAT2 mRNA were examined by qRT-PCR. The results are shown as fold change compared with Oil group mice. Data are the mean±SD of three independent experiments. ***$P < 0.001$, ****$P < 0.0001$, and ns, no significant.

Supplementary Figure S12

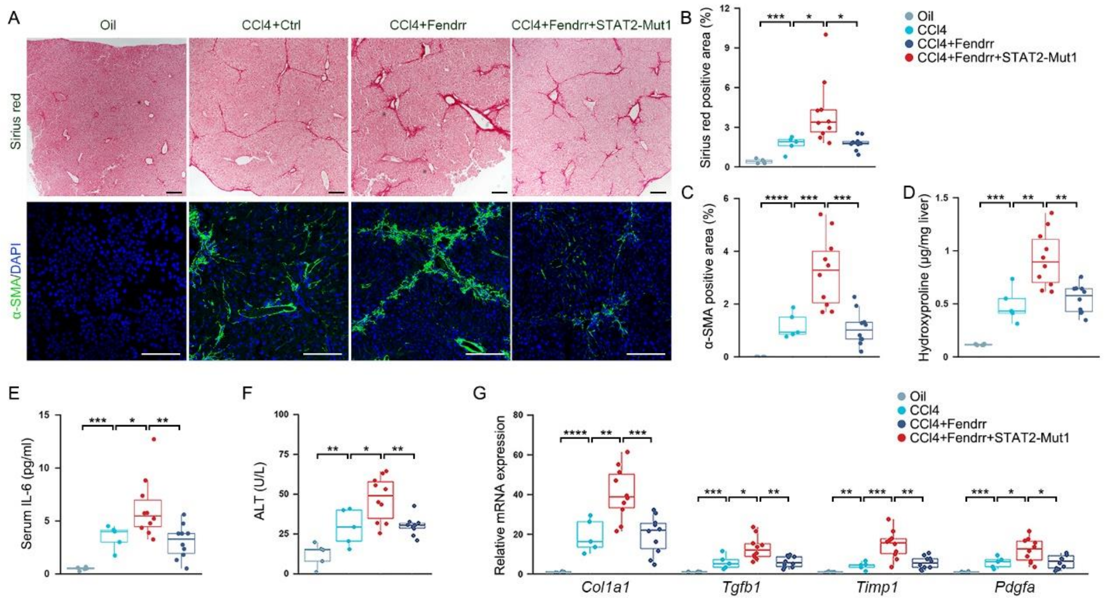

Supplementary Figure S12. Overexpression of STAT2 mutant abolished the profibrotic effect of Fendrr on CCl_4-induces liver fibrosis in mice. Mice were injected with oil (Oil, n=5), CCl_4 in combination with injection of control lentivirus (CCl4+Ctrl, n=5), CCl_4 in combination with injection of lv-Fendrr (CCl4+ Fendrr, n=10) and CCl_4 in combination with injection of lv-Fendrr and lv-STAT2-Mut1 (CCl4+Fendrr+STAT2-Mut1, n=10). The injection of lentivirus was started at two days before the first CCl_4 injection and last for four weeks at a frequency of twice per week. (A) The extent of liver fibrosis was evaluated by sirius red staining and α-SMA immunofluorescence staining. Scale bar, 100 μm. (B) Quantification of the sirius red positive area. (C) Quantification of α-SMA staining area. (D)Quantitative evaluation of hepatic hydroxyproline. The hydroxyproline contents are expressed as μg/mg wet liver weight. (E) Serum IL-6 was assayed by ELISA. (F) Assessment of serum ALT levels. (G) Hepatic *Tgfb1, Pdgfa, Col1a1* and *Timp1* mRNA expression were examined by qRT-PCR. The results are shown as fold change compared with Oil group mice. (H) Data are the mean±SD of at least three independent experiments. **P* < 0.05, ***P* < 0.01, ****P* < 0.001, *****P* < 0.0001.
